# Molecular Ionic Composite Polymer Electrolytes for High-Voltage Batteries

**DOI:** 10.1021/acsami.5c04566

**Published:** 2025-06-15

**Authors:** Jungki Min, Zhaohui Liang, Nicholas F. Pietra, Callum Connor, Louis A. Madsen, Feng Lin

**Affiliations:** † Department of Chemistry, 1757 Virginia Tech, Blacksburg, Virginia 24061, United States; ‡ Macromolecules Innovation Institute, Virginia Tech, Blacksburg, Virginia 24061, United States; § Department of Materials Science and Engineering, Virginia Tech, Blacksburg, Virginia 24061, United States

**Keywords:** solid-state lithium batteries, polymer electrolyte, Ni-rich cathode, interface stability, electrolyte additive

## Abstract

Polymer electrolytes are promising candidates for enabling safe, high-energy lithium batteries, particularly when paired with high-voltage layered oxide cathodes and lithium metal anodes. However, challenges at electrode|electrolyte interfaces, such as parasitic side reactions and electrolyte decomposition, have hindered the widespread adoption of polymer electrolyte-based high-voltage lithium batteries. To address these issues, this study introduces molecular ionic composites (MICs) as free-standing polymer electrolyte membranes, eliminating the need for any additional liquid electrolytes during cell assembly. MICs consist of a charged rigid-rod ionic polymer, poly-2,2″-disulfonyl-4,4′-benzidine terephthalamide (PBDT), combined with mobile ions from ionic liquids, lithium salts, and functional additives. The associative interactions between PBDT and these ions create a tunable platform with exceptional mechanical strength, moderate ionic conductivity, and enhanced electrochemical stability of polymer electrolyte over a wide temperature range. The optimized MIC electrolytes exhibit high ionic conductivity (3.21 mS cm^–1^ at 60 °C), a wide electrochemical stability window (5 V vs Li|Li^+^ based on linear sweep voltammetry), and excellent mechanical properties (tensile strength of 6.3 MPa, elastic modulus of 450 MPa). Furthermore, MICs enable good cycling stability in NMC811||Li metal cells, delivering an initial specific discharge capacity of 212 mAh g^–1^ and 93% capacity retention after 100 cycles at 2.8–4.4 V, C/3, and 60 °C. These results underscore the potential of MICs as a promising electrolyte platform for next-generation high-voltage lithium batteries and broader electrochemical energy storage applications.

## Graphical abstract

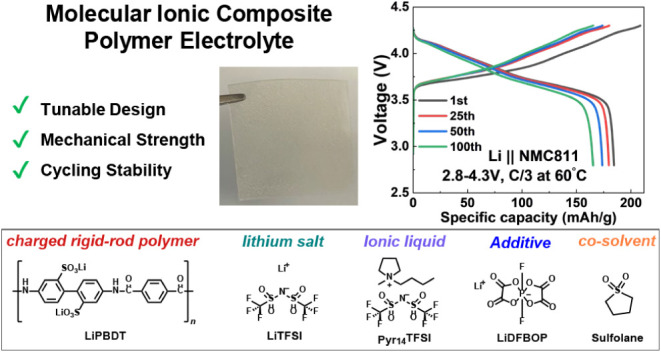

## 1 Introduction

Polymer electrolytes (PEs) have garnered attention as facilitators for high-energy-density solid-state batteries (SSBs) due to their excellent processability, malleability, and electrode conformability.^1–3^ However, combining PEs with a lithium metal anode and high-voltage cathodes to create SSBs presents many challenges, including the formation of lithium dendrite and the high-voltage instability of PEs at electrode–electrolyte interfaces.^4,5^ To tackle these issues, developing a materials platform to customize PEs is paramount to achieving high-voltage, solid-state lithium batteries.^6,7^

Molecular ionic composite (MIC) electrolytes are a distinct class of polymer electrolytes, characterized by collective associations derived from electrostatic interactions between charged, rigid-rod (and double-helical) ionic polymers (specifically poly-2,2′-disulfonyl-4,4′-benzidine terephthalamide, PBDT),^8,9^ and mobile ions originating from lithium salts and ionic liquids.^7,10–14^ These associative interactions of MICs provide robust mechanical properties and moderate ionic conductivity with a wide electrochemical stability window.^8,9^ Moreover, the enhanced safety features are endowed from nonflammable ILs, Li salts, and the aromatic polyamide PBDT, suggesting MICs as a promising choice for PEs in SSBs.^15^ MICs have the potential to serve as a versatile materials platform for solid-state electrolytes with a wide selection of components, such as lithium salts and ionic liquids, thereby offering a flexible solution to the challenges in high-voltage, solid-state lithium batteries.^16^

Sulfolane (SL) is a small molecule solvent with high oxidation stability (5.5 V vs Li|Li^+^), nonflammability, and a high dielectric constant (43.4).^17,18^ Moreover, SL has a high boiling point (285 °C) and lower viscosity than ILs, making it a suitable candidate for MIC components by forming a free-standing solid electrolyte membrane with decreased viscosity and free of solvent leakage.^19,20^ Also, the high dielectric constant of SL effectively dissolves lithium salts, which is conducive to incorporating functional additive salts into the MICs.

Herein, we report the rational design of MICs, combining SL to enhance transport and a functional salt additive to improve interfacial stability. This “gen 2 MIC” demonstrates good cycling performance in Li||LiNi_0.8_Co_0.1_Mn_0.1_O_2_ (NMC811) cells. Our work provides avenues for tailoring PEs to enable high-voltage lithium batteries.

## 2 Experimental Section

### 2.1 Materials

LiNi_0.8_Mn_0.1_Co_0.1_O_2_ (NMC811), characterized by secondary particle sizes around 5 μm and primary particles ranging from 200 to 500 nm in diameter, was sourced from the Cell Analysis, Modeling, and Prototyping (CAMP) Facility at Argonne National Laboratory. The lithium poly­(2,2′-disulfonyl-4,4′-benzidine terephthalamide) (LiPBDT) polymer was obtained from BlueSky Polymers and used without further purification. Reagents including sulfolane (≥99%), anhydrous *N*-methyl-2-pyrrolidone (NMP, 99.5%), and lithium bis­(trifluoromethanesulfonyl)­imide (LiTFSI, 99.99% trace metal basis, water content <250 ppm) were purchased from Sigma-Aldrich. The ionic liquid 1-butyl-1-methylpyrrolidinium bis­(trifluoromethylsulfonyl)­imide (Pyr_14_TFSI, ≥99.5%, water content 83 ppm) was supplied by Iolitec Inc. (Germany). Lithium difluorobis­(oxalate)­phosphate (LiDFBOP) with a purity of ≥99.9% and water content ≤500 ppm was acquired from Chemfish (Japan). Polyvinylidene fluoride (PVdF, ≥99% purity) and acetylene carbon black (≥99.8% purity, particle size 35–40 nm) were both sourced from MSE Supplies. For constructing pouch cells, lithium metal anodes were prepared using Cu–Li laminated foil, consisting of a 50 μm lithium layer on a 6 μm copper backing (90 mm wide, product code MA-EN-AN-000207, Canrd).

### 2.2 Preparation of Molecular Ionic Composite (MIC) Membrane

The gen 1 MIC electrolyte was synthesized following protocols established in prior studies.^14,16^ Both gen 1 and gen 2 MIC electrolytes were fabricated under ambient air conditions using benchtop procedures. For the gen 2 MIC formulation, 120 mg of LiPBDT was first dissolved in 12 g of high-purity water (HPLC grade, pH 7, resistivity >18 MΩ·cm at 25 °C; Fisher Scientific) using 6-dram glass vials with screw caps. Separately, a solution comprising 120 mg of LiTFSI, 360 mg of sulfolane, 36 mg of LiDFBOP, and 960 mg of Pyr_14_TFSI was prepared in 12 g of dimethylformamide (DMF, 99.7%, HPLC grade, Fisher Scientific) in identical vials.

Both mixtures were heated overnight at 80 °C in an Isotemp Model 280A vacuum oven (Fisher Scientific). The solutions were then combined, mixed thoroughly, and allowed to equilibrate at 80 °C overnight. The resulting homogeneous solution was cast onto a 10 × 10 cm^2^ glass substrate and left to dry at 80 °C under atmospheric pressure to remove residual solvents. To ensure complete drying, the membrane was subsequently placed in a vacuum oven (Isotemp Model 280A, Fisher Scientific) at 80 °C for an additional 48 h under a vacuum of 27 in. Hg.

After drying, the solidified gen 2 MIC membrane was detached from the glass plate using a cutter blade (18 mm, Bazik products) and cut into 19 mm diameter disks using a precision punch (MSK-T10, MTI). The membrane thickness was approximately 100 μm, measured using a digital thickness gauge (Mitutoyo 547-526S) with a force sensitivity of less than 1.5 nN.

### 2.3 Electrochemical Measurements

#### 2.3.1 Coin Cell Assembly and Testing

Cathodes were formulated by dispersing 92 wt % NMC811, 4 wt % polyvinylidene fluoride (PVdF) binder, and 4 wt % acetylene carbon black in *N*-methyl-2-pyrrolidone (NMP). The slurry was mixed using a SpeedMixer (DAC 150.1 FVZ-K, Hauschild) with a solvent-to-solid ratio of 0.5 mL NMP per 450 mg of total solids under ambient conditions. The homogenized slurry was then blade-coated (BYK doctor blade) onto a carbon-coated aluminum foil (1 μm dual-sided carbon coating on 15 μm Al foil, Canrd) and left to dry. Disks with a 10 mm diameter were punched from the coated foil and further dried in a vacuum oven (Vacutherm, ThermoFisher Scientific, model 51014551) overnight at 120 °C. These electrodes had an average thickness of 60 μm and a mass loading of 3.5 ± 0.5 mg cm^–2^.

Coin cells were assembled in an argon-filled glovebox (H_2_O, O_2_ < 0.5 ppm) using Li metal disks (15.6 mm diameter, 200 μm thick) as the counter electrodes. A stack pressure of 0.2 MPa was applied during assembly. Electrochemical characterization was performed with a Neware CT-4008T battery cycler, housed in a temperature-regulated chamber (DZF-6020, MTI).

Each electrochemical experiment included three cells, with data reported from a representative one. Specific capacity and current values were normalized to the active material mass in the cathode, based on the specific capacity of 200 mAh g^–1^ for NMC811. For lithium symmetric cells, the critical current density was evaluated through stepwise increases in current at 60 °C. Cycling tests for Li|MIC|NMC811 configurations were conducted between voltage cutoffs of 2.8–4.3 V or 2.8–4.4 V at 60 °C.

#### 2.3.2 Pouch Cell Fabrication and Testing

Single-layer Li∥NMC811 pouch cells were constructed using a stacked cell architecture with the molecular ionic composite (MIC) membrane serving as the solid electrolyte. The cathode consisted of LiNi_0.8_Mn_0.1_Co_0.1_O_2_ (NMC811) with an areal loading of approximately 3 mg cm^–2^. Electrode films were fabricated by casting a homogenized slurry, comprising 92 wt % NMC811, 4 wt % PVdF, and 4 wt % acetylene carbon black dispersed in NMP, onto carbon-coated aluminum foil (1 μm carbon layers on both sides of 15 μm aluminum foil, Canrd). Slurry mixing was performed using a SpeedMixer (DAC 150.1 FVZ-K, Hauschild), and film casting was conducted using a precision doctor blade (MTI) mounted on a TMAX-JK-TMJ-200 tape casting system (TMAX Scientific, China). After casting, electrodes were vacuum-dried at 120 °C overnight.

Anodes were cut from Cu–Li composite foils (90 mm wide, Canrd, product code MA-EN-AN-000207), composed of 50 μm lithium laminated onto a 6 μm copper substrate, and trimmed into 3 × 5 cm^2^ rectangles. MIC electrolyte membranes, approximately 100  μm in thickness, were prepared and trimmed into 3.3 × 3.3 cm^2^ squares to serve as separators.

Cell assembly was conducted inside an argon-filled glovebox (H_2_O, O_2_ < 0.5 ppm). The components were stacked in the following order: lithium anode, MIC membrane, and NMC811 cathode. The lithium foil was adhered to the pouch cell casing using Kapton tape before layering the electrolyte and cathode. Current collectors (nickel for the anode and aluminum for the cathode) were attached via spot-welding. The final cell stack was sealed using a heat sealer at approximately 180 °C. A uniform pressure of ∼1 MPa was applied using a custom-designed clamp to ensure consistent interfacial contact during operation.

Electrochemical cycling was performed using a Neware CT-4008T tester inside a temperature-controlled chamber (DZF-6020, MTI). After resting for 24 h, the cells were cycled galvanostatically within a voltage window of 2.8–4.4 V at 60 °C. Capacity and current density values were normalized to the NMC811 cathode’s active material mass, based on a specific capacity of 200 mAh g^–1^. Care was taken to maintain proper alignment and ensure complete interfacial coverage of the MIC membrane for reproducible performance.

#### 2.3.3 Measurement of Transference Number (*t*_Li^+^_)

The lithium-ion transference number (*t*__Li_^+^_) was evaluated via potentiostatic polarization using a BioLogic SP-200 electrochemical workstation. Lithium symmetric cells were applied with a constant direct current voltage bias of 10 mV (Δ*V*) at 23 °C. Electrochemical impedance spectroscopy (EIS) was carried out both prior to and following polarization across a frequency range of 5 MHz to 0.1 Hz, with 100 data points collected per frequency decade. Prior to initiating measurements, the system was allowed to stabilize at its open-circuit voltage. The transference number was then computed based on the following expression:^21^

1 $$t_{{\mathrm{Li}}^{+}}=\frac{I_{\mathrm{ss}}\left(\Delta V-I_{0}R_{0}\right)}{I_{0}\left(\Delta V-I_{\mathrm{ss}}R_{\mathrm{ss}}\right)}$$

*I*_0_ denotes the initial current response immediately after applying the voltage step, while *I*_ss_ represents the steady-state current measured after 1 h. *R*_0_ corresponds to the cell’s initial interfacial resistance, and *R*_ss_ is the resistance at steady state, both determined from EIS measurements.

#### 2.3.4 Ionic Conductivity Measurement

Symmetric cells were assembled using stainless steel (304 SS) electrodes, each 1 mm thick with a 15.8 mm diameter (Canrd), in an argon-filled glovebox (H_2_O, O_2_ < 0.5 ppm). The molecular ionic composite (MIC) membrane, approximately 100 μm thick, was placed between the two electrodes and compressed under a pressure of 0.2 MPa. The stainless-steel contact area was 16 mm in diameter. The assembled cells were equilibrated overnight at 60 °C before measurements. Electrochemical impedance spectroscopy (EIS) was performed once per sample using a potentiostatic method, sweeping frequencies from 1 Hz to 1 MHz, with 100 points collected per frequency decade. The ionic conductivity (σ) was determined using the following relation:

2 $$\sigma =\frac{d}{R_{b}\times S}$$

In this equation, *d* represents the thickness of the MIC membrane, *R*_b_ is the bulk resistance extracted from the high-frequency intercept of the Nyquist plot, and *S* denotes the contact area of the stainless-steel electrodes.

#### 2.3.5 Linear Sweep Voltammetry (LSV) Measurement

To determine the electrochemical stability window of the MIC membrane, linear sweep voltammetry was performed using a Biologic SP150 potentiostat. The measurement was conducted at 60 °C within a temperature-controlled chamber. Aluminum foil served as the working electrode, while lithium metal was employed as both the counter and reference electrodes. The potential was swept at a scan rate of 0.1 mV s^– 1^.

### 2.4 Physicochemical Characterization

The Li anode and NMC cathode surface from cycled Li|MIC|NMC samples are characterized using a scanning electron microscope (SEM, JEOL IT500). The thermal and mechanical properties of the gen 2 MIC are measured by dynamic mechanical thermal analysis (DMTA). Thermogravimetric (TGA) analysis is conducted to evaluate the thermal stability of the gen 2 MIC membrane. The nuclear magnetic resonance (NMR) diffusometry measurements are performed to measure the diffusion coefficient of electrolyte membranes with temperature variation. The chemical composition of the cycled MIC membrane was quantitatively analyzed using X-ray photoelectron spectroscopy (XPS) on a PHI VersaProbe III system, equipped with a monochromatic Al Kα X-ray source operating at 1486.6 eV.

#### 2.4.1 Mechanical Property Characterization

Mechanical testing of the gen 2 MIC membrane was performed using a TA Instruments DMA 850, fitted with a film tension clamp, to conduct uniaxial tensile measurements. The measurements were performed at 30 °C under a constant loading rate of 1  N min^– 1^ until failure. The membrane exhibited an average tensile strength of 6.3 MPa and an average elastic modulus of 450 MPa from three independent measurements. These values were determined from the stress–strain curve obtained during the test. The measured elastic modulus of the gen 2 MIC is consistent with previously reported values for MIC membranes containing 10 wt % PBDT, as documented in our earlier work.^11,14^

#### 2.4.2 Thermal Gravimetric Analysis (TGA)

Thermal stability of the gen 2 MIC membrane was evaluated via thermogravimetric analysis (TGA) using a TA Instruments TGA550 system. Measurements were conducted in a dry nitrogen environment with platinum sample pans, applying a heating rate of 10 °C min^– 1^ throughout the analysis. TGA measurements were also carried out on individual components of the MIC, including LiDFBOP, sulfolane, LiTFSI, Pyr_14_TFSI, and LiPBDT.

#### 2.4.3 NMR Diffusometry Measurements

MIC membranes were loaded into 5 mm NMR tubes, dried at 100 °C under vacuum using a hot sand bath, and subsequently flame-sealed under vacuum conditions in preparation for diffusion measurements. The NMR spectra and corresponding diffusion data were acquired using a Bruker Avance III 400 MHz (9.4 T) wide-bore spectrometer, equipped with a high-gradient diffusion probe (Bruker Diff50) and a 5 mm ^1^H RF coil insert.

Diffusion measurements were carried out across a temperature range of 25 to 80 °C, with each temperature point preceded by at least 5 min of thermal equilibration. The pulsed-gradient stimulated echo (PGSTE) method was used to probe the self-diffusion behavior of cations and anions via ^1^H and ^19^F NMR, respectively. The Stejskal-Tanner equation was applied to model the signal intensity (*I*) as a function of gradient strength (*g*):

3 $$I=I_{0}\exp\left(-D\gamma^{2}g^{2}\delta^{2}\left(\Delta -\frac{\delta }{3}\right)\right)$$

In this equation, *I*_0_ represents the signal intensity at zero gradient strength (*g* = 0), γ is the gyromagnetic ratio of the observed nucleus, δ denotes the duration of the gradient pulse, Δ is the time interval between the gradient pulses, and *D* refers to the self-diffusion coefficient. The experimental parameters included a 90° pulse width of 3.7 μs for ^1^H and 6 μs for ^19^F nuclei, with a uniform acquisition time of 0.02 s. Diffusion measurements were carried out with repetition delays between 1 and 2 seconds, using δ = 1 ms and Δ = 50 ms. Gradient strengths were varied from 600 to 1500 G/cm across 16 steps to ensure a minimum of 85% signal attenuation.

#### 2.4.4 X-Ray Photoelectron Spectroscopy

The XPS binding energy scale was calibrated using the aliphatic carbon (C 1s) peak positioned at 284.8 eV. Following electrochemical cycling, Li|MIC|NMC811 coin cells were disassembled inside an argon-filled glovebox, where moisture and oxygen levels were maintained below 0.5 ppm. The cathode (NMC811) and anode (lithium metal) were collected, gently rinsed with dimethyl carbonate (purity ≥99.9%, water content <10 ppm, Sigma-Aldrich) to remove residual ionic liquid, and then dried briefly within the glovebox before being sealed in transfer containers.

Each sample was mounted onto the XPS sample holder using double-sided adhesive tape. Surface analysis was carried out at the University of Delaware using a PHI VersaProbe III spectrometer (Physical Electronics, MN, USA), which employed a monochromatic Al Kα X-ray source operating at 50 W with an energy of 1486.6 eV. The scanned area measured 1000 by 200 μm. Peak fitting and elemental quantification were performed using PHI MultiPak Software Version 9.9.0.8, applying a Shirley background and a combined Gaussian–Lorentzian peak shape. Sensitivity factors used for quantification were internally corrected by the software.

## 3 Results and Discussion

### 3.1 Characterization of Molecular Ionic Composite Electrolytes

The previous “gen 1” MIC electrolyte membrane consists of 10 wt % PBDT polymer with 10 wt % lithium bis­(trifluoromethyl sulfonyl)­imide (LiTFSI), 80 wt % 1-butyl-1-methylpyrrolidinium bis­(trifluoromethyl sulfonyl)­imide (Pyr_14_TFSI).^14^ Building upon the gen 1 MIC platform, the gen 2 MIC presented here offers a facile yet effective enhancement through the incorporation of sulfolane and LiDFBOP, enabling synergistic improvements in oxidative stability, interphase chemistry, and diffusion without relying on ceramic fillers. In this work, the gen 2 MIC electrolyte membrane is composed of 7.5 wt % PBDT polymer with 7.5 wt % lithium bis­(trifluoromethyl sulfonyl)­imide (LiTFSI), 60 wt % 1-butyl-1-methylpyrrolidinium bis­(trifluoromethyl sulfonyl)­imide (Pyr_14_TFSI), 22.5 wt % of sulfolane (SL), and 2.5 wt % of lithium difluorobis­(oxalate) phosphate (LiDFBOP) as shown in (Figure 1a). LiDFBOP was introduced into the MIC formulation as a functional additive to improve electrochemical cycling stability.^22–25^ The free-standing gen 2 MIC membrane is prepared from solvent casting (Figure 1b). The SEM image shows the homogeneous surface of the as-prepared membrane without any salt agglomeration (Figure 1c). Figure 1d shows the uniaxial tensile stress–strain curve of the gen 2 MIC membrane measured at 30 °C. The membrane exhibited an average tensile strength of 6.3 MPa and an elastic modulus of 450 MPa, based on three independently measured trials, reflecting mechanical robustness suitable for handling and integration in cell assembly. These values reflect robust mechanical integrity compared to previously reported polymer electrolytes (Table S1).^11–13^ Thermal stability was assessed through thermogravimetric analysis (TGA), performed from 30 °C to 650 °C at a heating rate of 10 °C per minute, as shown in Figure 1e. The gen 2 MIC membrane exhibited an initial mass loss of ∼5% below 200 °C, followed by a gradual loss totaling ∼20% up to 400 °C, and substantial decomposition beyond this temperature. To help interpret these weight losses, TGA was also performed on the individual MIC components, including LiDFBOP, sulfolane, LiTFSI, Pyr_14_TFSI, and LiPBDT (Figure S1). The initial ∼5% loss below 200 °C is attributed to the decomposition of SL (onset at ∼170 °C) and LiDFBOP (onset at ∼150 °C), while the additional mass loss up to 400 °C reflects the progressive thermal degradation of the MIC constituents (Figures 1e and S1). To further evaluate the membrane’s stability under prolonged high-temperature exposure, isothermal TGA measurements were conducted at 300, 350, and 400 °C, simulating thermal abuse conditions relevant to battery failure scenarios (Figure S2).

**Figure 1:**
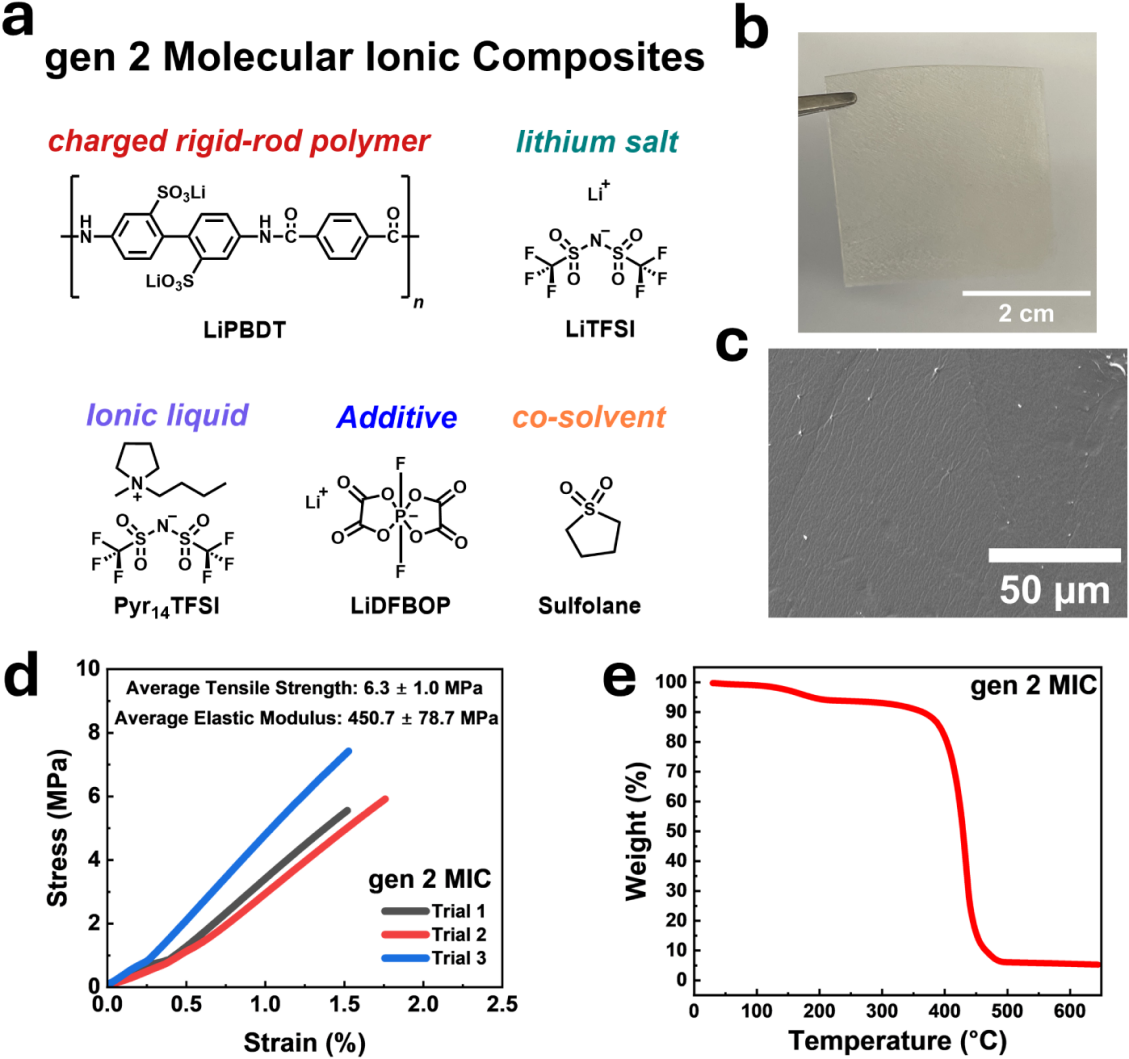
Chemical structure and characterization of the gen 2 MIC electrolytes. (a) Chemical ingredients of the gen 2 MIC electrolytes. (b) Digital image of free-standing gen 2 MIC electrolyte membrane. (c) SEM image of as-cast MIC membrane. (d) Stress–strain curves of gen 2 MIC membrane at 30 °C, showing results from three independent measurements. (e) Thermogravimetric analysis (TGA) of gen 2 MIC membrane at a heating rate of 10 °C min^–1^ in nitrogen atmosphere, showing minimal weight loss up to 400 °C. TGA profiles of the individual components and isothermal TGA of gen 2 MIC are provided in Figures S1 and S2.

As shown in Figure 2a, the gen 2 MIC electrolyte exhibits ionic conductivities of 0.66 mS cm^–1^ at 23 °C and 3.21 mS cm^–1^ at 60 °C. The lithium-ion transference number at 60 °C is measured to be 0.2, as illustrated in Figure 2b. Linear sweep voltammetry (LSV) results at 60 °C for both the gen 1 MIC and the gen 2 MIC are presented in Figure 2c. For the gen 1 MIC, an oxidative current peak is observed around 3.4 V vs Li|Li^+^, with a corresponding current of 0.1 mA, which is attributed to the oxidative degradation of the PBDT polymer.^14^ Meanwhile, the gen 2 MIC shows improved stability against oxidative decomposition, ascribed to the passivation layer from sulfolane and LiDFBOP.^22,25–27^ The self-diffusion coefficients of IL are measured by pulsed-field-gradient (PFG) NMR diffusometry to explore the ion transport behavior of gen 1 MIC and gen 2 MIC.^10^ Figure 2d presents the self-diffusion coefficients of cations (D^+^) and anions (D^–^), measured using ^1^H and ^19^F NMR, respectively, over a temperature range of 23 to 80 °C. The values are averaged across the full thickness of the electrolyte membrane. In both gen 1 and gen 2 MIC electrolytes, cation diffusion is consistently faster than anion diffusion across the entire temperature range. The total ionic diffusivity (D, the summation of the cation and anion diffusion coefficients) of the gen 1 MIC and the gen 2 MIC are 2.59 × 10^–11^ and 3.39 × 10^–11^ m^2^/s, respectively, at 60 °C, which is 31% higher for the designed MIC. Overall, the gen 2 MIC shows improved oxidation stability and a higher diffusion coefficient than the gen 1 MIC from the compositional tailoring. These comparisons between the gen 1 and gen 2 MIC electrolytes are tabulated in Figure S3. While sulfolane (SL) and LiDFBOP have each been studied individually in conventional liquid electrolyte systems, to our knowledge, this work presents the first demonstration of their integration into a charged rigid-rod ionic polymer matrix based on poly­(2,2″-disulfonyl-4,4′-benzidine terephthalamide) (PBDT). Compared to gen 1, the gen 2 MIC exhibits enhanced ionic conductivity, higher ionic diffusivity, improved oxidative stability, and comparable mechanical properties (tensile strength and elastic modulus). These property gains contribute to improved cycling stability and interfacial behavior, as demonstrated in the following sections.

**Figure 2:**
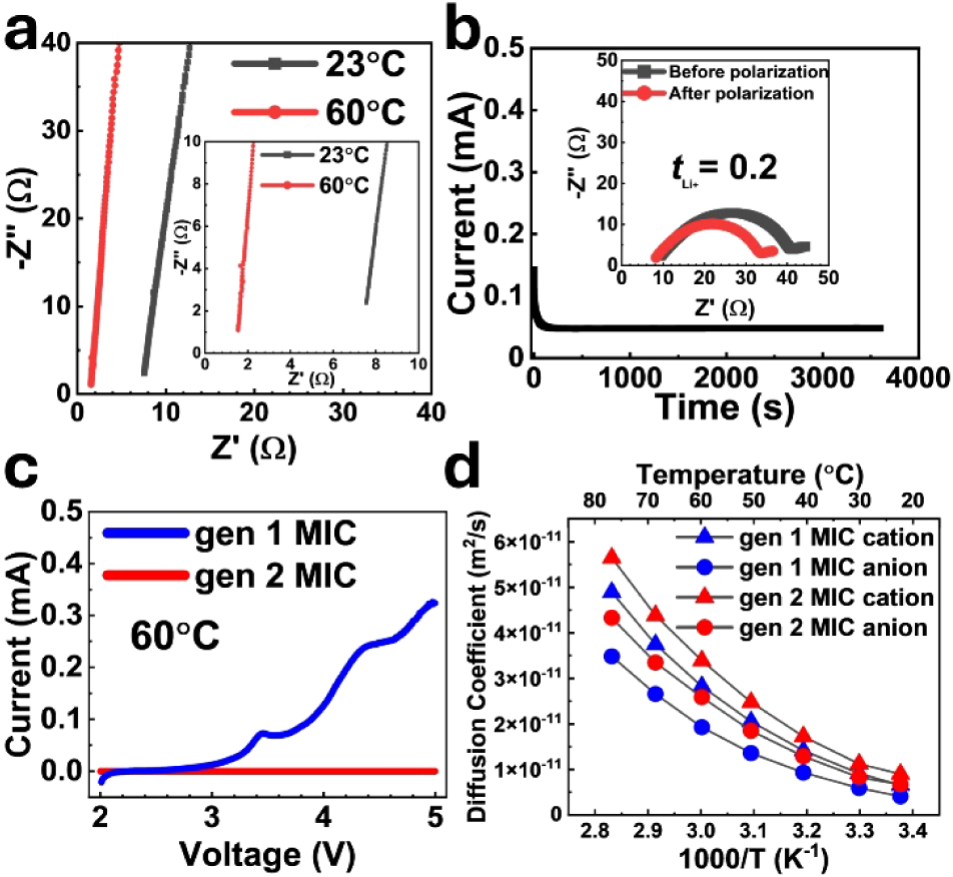
Electrochemical characterization and NMR diffusometry measurement of gen 2 MIC electrolytes. (a) Electrochemical impedance spectroscopy (EIS) measurements. The inset shows enlarged view of graph. (b) Chronoamperometry profile. (c) Linear sweep voltammetry (LSV) plots. (d) NMR diffusometry results. The diffusion coefficient of the gen 1 MIC is provided to showcase that the gen 2 MIC has superior diffusion properties.

### 3.2 Electrochemical Performance of Molecular Ionic Composite Electrolytes

In Figure 3a, Li symmetric cells are cycled to evaluate electrolyte compatibility with the Li metal anode at 60 °C under increasing stepped current density. The gen 1 MIC limiting current density is 0.65 mA cm^–2^, while the gen 2 MIC achieves 0.95 mA cm^–2^ (Figure 3a). The decreased overpotential and higher limiting current density of the gen 2 MIC suggest enhanced lithium plating and stripping performance. The inset of Figure 3a shows overpotentials of 184 mV and 121 mV for gen 1 and gen 2 MICs, respectively. Figure 3b shows a Li symmetric cell using the gen 2 MIC electrolyte cycled at 0.3 mA cm^–2^ and 60 °C for 500 h, exhibiting relatively stable cycling behavior, though a gradual increase in overpotential is observed over time (∼320 mV at 500 h).

**Figure 3:**
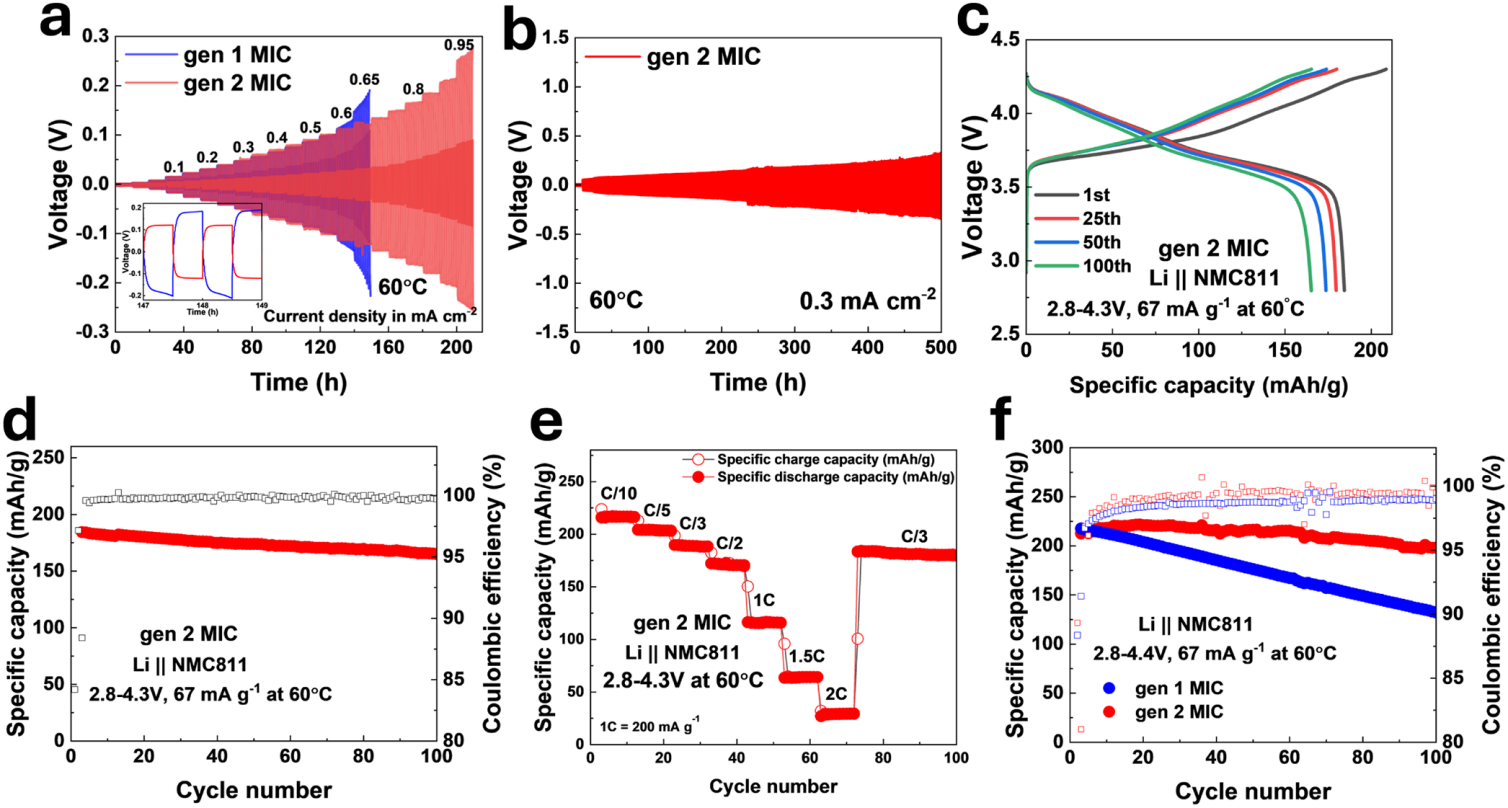
Performance of Li||Li symmetric cells and Li||NMC811 cells. (a) Voltage profiles of Li symmetric cells using gen 1 and gen 2 MIC electrolytes at 60 °C under stepwise increases in current density. Each charge and discharge step lasted 0.5 hours, with the current density increasing every 10 cycles. The inset displays an enlarged view of the profiles between 147 and 149 h. The gen 2 MIC exhibits a higher limiting current density and lower overpotential. (b) Long-term cycling performance of a Li symmetric cell with gen 2 MIC at 60 °C under a constant current density of 0.3 mA cm^–2^. The cell was initially conditioned with 10 cycles at 0.05 mA cm^–2^. (c) Voltage profiles at the first, 25th, 50th, and 100th cycles (2.8–4.3 V, 67 mA g^–1^, 60 °C). (d) Cycling stability at 2.8–4.3 V, 67 mA g^–1^, and 60 °C. (e) Rate capability at 2.8–4.3 V and 60 °C. (f) Extended cycling at 2.8–4.4 V, 67 mA g^–1^, and 60 °C. The gen 2 MIC displays significantly improved cycling stability compared to gen 1. All cells underwent two formation cycles at 10 mA g^–1^ prior to the measurements shown in panels (c) through (f).

To assess the electrochemical performance of the gen 2 MIC electrolyte, Li|MIC|LiNi_0.8_Co_0.1_Mn_0.1_O_2_ (NMC811) cells were assembled and cycled at 60 °C with upper cutoff voltages of 4.3 and 4.4 V. This temperature was selected to balance enhanced ion mobility, facilitated by the reduced viscosity of the ionic liquid in the MIC matrix, with the thermal sensitivity of Ni-rich NMC811 cathodes, which are prone to degradation at elevated temperatures. Figure 3c shows a representative voltage profile at 60 °C with a cutoff of 4.3 V over 100 cycles, delivering an initial discharge specific capacity of 184 mAh g^–1^ and retaining 165 mAh g^–1^ after 100 cycles (Figure 3d). Stable performance is also observed under varying current densities (Figure 3e), with capacities of 172 mAh g^–1^ at C/2 and 116 mAh g^–1^ at 1C. Figure 3f shows comparative cycling between gen 1 and gen 2 MICs at 4.4 V and 60 °C, with gen 2 MIC achieving a discharge capacity of 212 mAh g^–1^ and 93% retention after 100 cycles, whereas gen 1 MIC shows 61% retention.

To further evaluate near-ambient temperature performance, Li∥gen 2 MIC∥NMC811 cells were cycled at 35 °C and 0.2C (40 mA  g^–1^) between 2.8–4.4 V. As shown in Figure S4, the cell delivered 181 mAh  g^–1^ by the sixth cycle and exhibited stable capacity over 50 cycles. The initial capacity of 156 mAh g^–1^ gradually increased during early cycles, likely due to progressive wetting of the porous cathode by the viscous liquid phase in MIC.

We further assembled single-layer Li|gen 2 MIC|NMC811 pouch cells, which were then cycled at 60 °C. The pouch cell consisted of an NMC811 cathode (areal capacity ∼0.6 mAh cm^–2^), a 50 μm-thick lithium metal anode, and the gen 2 MIC electrolyte. After two initial preconditioning cycles at 0.05 C (10 mA g^–1^), the pouch cell delivered a specific discharge capacity of 178 mAh g^–1^ at 67 mA g^–1^ and exhibited stable cycling performance over 20 cycles (Figure S5).

Figure 4a,b shows the scanning electron microscopy (SEM) image of the cycled lithium metal anode and NMC811 cathode, interfaced with gen 2 MIC, after 100 cycles with an upper cutoff voltage of 4.4 V at 60 °C. The uniform plating of Li on the Li anode surface is observed in Figure 4a, which aligns with the efficient plating/stripping voltage profile in Figure 3a,b. The results indicate that gen 2 MIC has improved lithium plating/stripping and good dendrite suppression. The surface of the cycled NMC811 cathode shows uniform coverage of gen 2 MIC over active material (Figure 4b), suggesting intimate contact between gen 2 MIC and electrode. The Nyquist plot of gen 2 MIC is shown in Figure 4c, with a stable resistance profile up to 100th cycles. The decrease in the interfacial resistance after the 100th cycle (Figure 4c) suggests an intimate contact and stable passivation layer is formed between electrodes and electrolytes,^22,28^ which aligns with Li||NMC811 cell cycling results.

**Figure 4:**
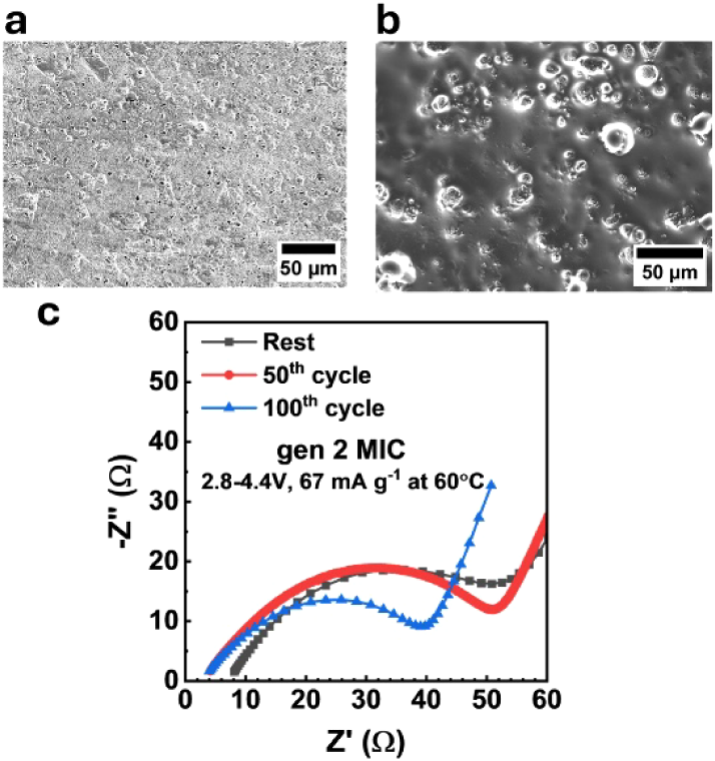
SEM images of the electrode surface and impedance spectra of Li||NMC811 cells. The cell is cycled under 2.8–4.4 V, 67 mA g^–1^ at 60 °C. The SEM images are collected from electrode samples of cells after 100 cycles, and impedance is measured after a specific number of cycles. SEM image of (a) lithium metal surface and (b) NMC811 cathode surface. The bright regions on the NMC811 cathode surface correspond to active materials, while the darker areas indicate the MIC electrolyte coating the cathode. (c) Impedance spectra.

### 3.3 Compositional Analysis of electrode|electrolyte Interface

Based on the improved cell performance of gen 2 MIC electrolyte, XPS analysis is conducted to understand the chemical composition of the passivation layer formed at the electrode|electrolyte interfaces. Figure 5 shows the XPS analysis results of the surface of NMC811 and Li metal electrodes from gen 2 MIC-based coin cell after electrochemical cycling (Li||NMC811, 2.8–4.4 V, 67 mA g^–1^, 200 cycles at 60 °C).

**Figure 5:**
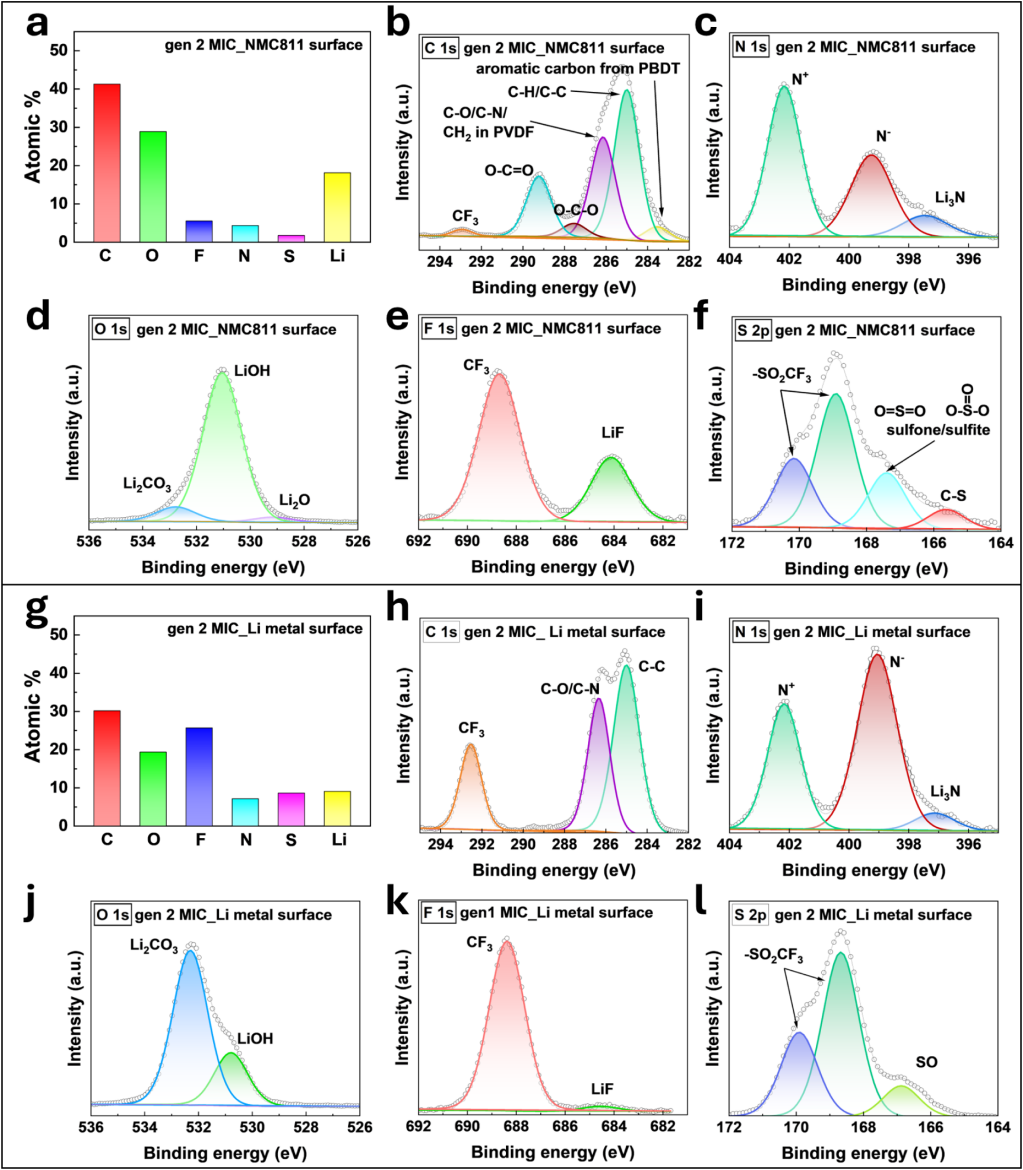
XPS analysis of NMC811 and Li metal surface from gen 2 MIC-based coin cell after electrochemical cycling (2.8–4.4 V, 67 mA g^–1^, 200 cycles at 60 °C). The XPS results of the NMC811 surface are shown in the left panel from (a) to (f). Meanwhile, the XPS results of the Li metal surface are presented in the right panel from (g) to (l). (a) Atomic concentration of elements on NMC811 surface: C (carbon), O (oxygen), F (fluorine), N (nitrogen), S (sulfur), and Li (lithium). Trace amounts (0.05%) of phosphorus (P) element are also observed. The phosphorus originates from the decomposition of LiDFBOP (Lithium difluorobis­(oxalate)­phosphate). Specific XPS spectra of NMC811 surface are shown in (b) C 1s, (c) N 1s, (d) O 1s, (e) F 1s, (f) S 2p. From C 1s spectra, sp^2^ carbon is denoted as “aromatic carbon from PBDT.” (g) Atomic concentration of elements on Li metal surface: C (carbon), O (oxygen), F (fluorine), N (nitrogen), S (sulfur), and Li (lithium). Specific XPS spectra of Li metal surface are shown in (h) C 1s, (i) N 1s, (j) O 1s, (k) F 1s, (l) S 2p. From N 1s spectra of (c) and (i), N^+^ and N^–^ originate from ionic liquid, i.e., pyrrolidinium cation (Pyr_14_^+^) and (trifluoromethanesulfonyl)­imide anion (TFSI^–^), respectively.

Lithium nitride (Li_3_N)^29,30^ and sulfurous compounds (SO, C–S)^31–33^ are observed in the NMC811 surface and Li metal surface of the gen 2 MIC-based cells (Figure 5c,f,i,l). These are reported to form a beneficial interphase, enabling stable cycling performance.^34–38^ Lithium nitride stems from the decomposition of TFSI^–^ (from LiTFSI and Pyr_14_TFSI) in gen 2 MIC electrolyte, which is promoted by the synergistic effect of additives in gen 2 MIC (LiDFBOP, sulfolane).^39–41^ The sulfurous compounds (SO, C–S) are from the decomposition of sulfolane.^27,42^ The formation of lithium nitride (Li_3_N) and from the decomposition of LiTFSI is reported in both cathode^43,44^ and Li metal^45,46^ previously. In addition, the formation of sulfurous compound (C–S) from sulfolane decomposition at high-voltage cathode^47^ and (SO) at Li metal^33^ is previously studied, which aligns with our observation in Figure 5f and l.

Figure 5b shows O–C=O and O–C–O peaks derived from the decomposition of the oxalate group of LiDFBOP, indicating coverage of the NMC811 surface with an additive from gen 2 MIC. Moreover, low intensity of sp^2^ C peaks labeled as “aromatic carbon from PBDT” is observed in Figure 5b, suggesting that the passivation layer from additives minimizes the exposure of PBDT (polymer) from oxidative degradation. LiDFBOP readily reacts on electrode surface to passivate the interfaces, while facilitating the decomposition of LiTFSI^40^ and sulfolane to form a beneficial interphase (i.e., Li_3_N and sulfurous compounds). The additives play a crucial role in governing the local environment at interfaces after its decomposition and influence the subsequent reactions,^40^ impacting the electrochemical performance of batteries. We hypothesize that additives in gen 2 MIC delay architectural support (PBDT polymer) degradation in polymer electrolytes, leading to improved cycling stability, as shown in Figure 3f. The gen 1 MIC shows a higher intensity of sp^2^ C peaks from PBDT, suggesting that additives in gen 2 MIC passivate the electrode|electrolyte interfaces (XPS spectra of electrodes surface from gen 1 MIC cells are provided in Figures S6 and S7).

The phosphorus (P) element from the LiDFBOP additive does not show significant signal intensity that can be analyzed, presumably due to the low atomic ratio of the P element in the LiDFBOP molecule and the small amount added to the gen 2 MIC electrolyte. Moreover, decomposition products of LiDFBOP (e.g., Li_*x*_PO_*y*_F_*z*_·Li_2_C_2_O_4_) overlap with other peaks in O 1s and F 1s spectra.^41,48^

From the XPS analysis in Figure 5, the C 1s spectra are assigned as CF_3_ (293 eV), CO_3_^2–^ (290 eV), O–C=O (289 eV), O–C–O (287 eV), C–O (286.1 eV), C–N (286 eV), CH_2_ in PVDF (286.2 eV), C–C (285 eV), C–H (∼285 eV), and sp^2^ C (283.5 eV).^41,45,49,49–52^ N 1s spectra are assigned as N^+^ (402 eV), N^–^ (399 eV) from pyrrolidinium cation (Pyr_14_^+^) and (trifluoromethanesulfonyl)­imide anion (TFSI^–^), respectively.^45^ Moreover, from N 1s spectra in Figure 5c and i, Li_3_N (397 eV) is also observed in gen 2 MIC electrolyte-based Li||NMC811 cells.^53^ O 1s spectra in Figure 5d,j show Li_2_CO_3_ (532.5 eV), LiOH (531 eV), and Li_2_O (528 eV).^45,52,54^ In Figure 5e,k, the F 1s spectra show peaks assigned to CF_3_ (688 eV) and LiF (684 eV).^45^ S 2p spectra in Figure 5f,l show characteristic peaks ascribed to −SO_2_CF_3_ (doublet 169 eV, 170 eV) from TFSI^–^, sulfone/sulfite (167 eV), SO (166.8 eV), and C–S (165 eV).^33,45^

### 3.4 Comparison Plot of Molecular Ionic Composites with Polymer Electrolytes

Figure 6 shows the comparison plot of gen 2 MIC with recent reports of polymer electrolytes paired with high-voltage layered oxide cathode (e.g., LiNi_0.8_Mn_0.1_Co_0.1_O_2_ (NMC811)) and Li metal. The legend’s name is based on cited literature, indicating which base polymer is used in making polymer electrolytes. The numerical values are provided in Table S1. “This work” represents the properties of gen 2 MIC in this manuscript. “Our previous work” is on gen 1 MIC.^14^ PVDF–LLZO stands for poly­(vinylidene fluoride) (PVDF) integrated with inorganic filler Li_7_La_3_Zr_2_O_12_ (LLZO).^55^ PEU–MOF indicates poly­(ether-urethane) incorporated with a metal–organic framework (MOF).^56^ PPES stands for poly­[poly­(ethylene glycol) methyl ether methacrylate)-*r*-(2-ethylhexyl acrylate)-*r*-sodium (*p*-styrenesulfonate)-*r*-polyethylene glycol dimethacrylate] (PPES).^57^ PVDF–HFP–IL indicates poly­(vinylidene fluoride-*co*-hexafluoropropylene) (PVDF–HFP) integrated with ionic liquid.^58^ polyDOL@SEP represents polymerized 1,3-dioxolane (DOL) supported on the separator.^59^ PEO-based means poly­(ethylene oxide)-based polymer electrolyte.^60^ PVDF–HFP-based@PE indicates poly­(vinylidene fluoride-*co*-hexafluoropropylene) (PVDF–HFP) supported on polyethylene (PE).^61^ polyDOL–PVDF–LLZTO represents poly-1,3-dioxolane in a poly­(vinylidene fluoride)/Li_6.4_La_3_Zr_1.4_Ta_0.6_O_12_ (LLZTO) matrix.^62^ PCL-based stands for poly­(ε-caprolactone) (PCL)-based polymer electrolyte.^63^ PCL–LATP indicates poly­(ε-caprolactone) (PCL) incorporated with Li_1.4_Al_0.4_Ti_1.6_(PO_4_)_3_ (LATP).^64^

**Figure 6:**
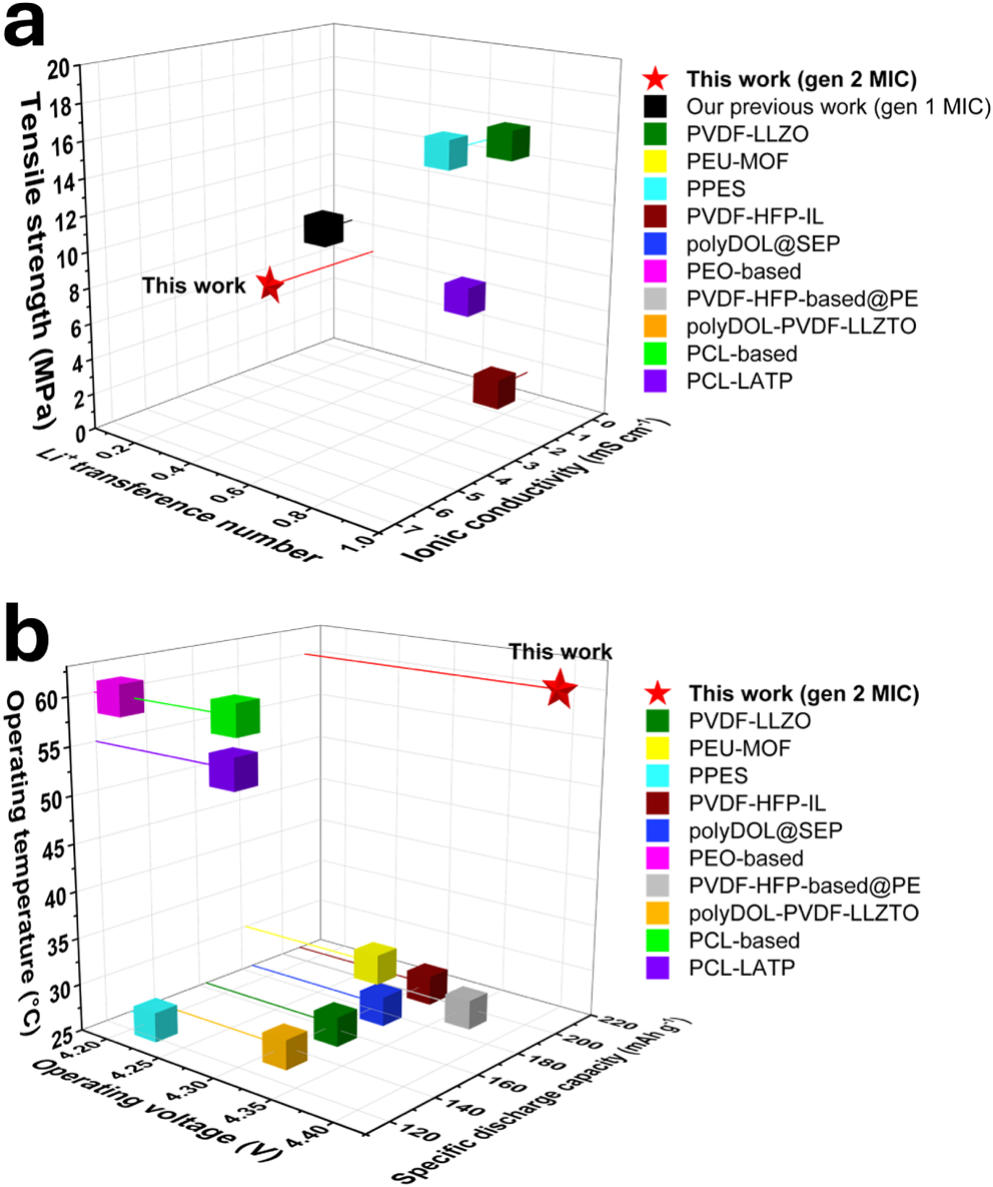
Comparison of gen 2 MIC with other reported polymer electrolytes in terms of physical properties, electrochemical characteristics, and cell performance. The selected examples involve polymer electrolytes paired with common cathode materials in lithium batteries, including LiNi_0.8_Mn_0.1_Co_0.1_O_2_ (NMC811), LiFePO_4_ (LFP), LLiNi_0.8_Mn_0.1_Co_0.1_O_2_ (NMC622), and LiNi_0.8_Mn_0.1_Co_0.1_O_2_ (NMC523). (a) 3D plot of Li^+^ transference number (*X*-axis), ionic conductivity (mS cm^–1^, *Y*-axis), and tensile strength (MPa, *Z*-axis). (b) 3D plot of operating voltage (*X*-axis), specific discharge capacity (mAh g^–1^, *Y*-axis), and operating temperature (°C, *Z*-axis). Legends indicate the base polymer types used in each system. Detailed data are provided in Table S1.

From Figure 6a, gen 2 MIC (this work) shows good tensile strength and moderate ionic conductivity, albeit a low Li^+^ transference number. The mechanical properties (tensile strength of 6.3 MPa, elastic modulus of 450 MPa) of the gen 2 MIC allow free-standing polymer electrolyte membranes without incorporating inorganic fillers or support from a commercial polyethylene (PE) separator, which is typical for state-of-the-art polymer electrolytes (Table S1).^55,56,61,62,64^ The moderate tensile strength and high elastic modulus provide mechanical properties suitable for a free-standing polymer electrolyte membrane. Moreover, MIC electrolyte membranes do not require an Ar-filled glovebox to prepare polymer electrolyte membranes. They are benchtop stable and absent from complex synthesis procedures, unlike other polymer electrolytes, which necessitate an inert environment and multiple steps for electrolyte preparation.^55,56^ MIC functions as a solid-state polymer electrolyte membrane, eliminating the need for an additional liquid electrolyte soaking process before cell assembly, unlike other polymer electrolyte systems.^57^ As illustrated in Figure 6b, the gen 2 MIC in this study demonstrates potential for operation under elevated-temperature and high-voltage conditions, delivering a higher specific discharge capacity than many reported polymer electrolytes. Moreover, gen 1 MIC showed Li||LiFePO_4_ cell cycling at 150 °C with outstanding electrochemical performance (2.7–4.2 V, 400 cycles, 160 mAh g^–1^ at 1C and 150 °C).^14^

## 4 Conclusions

In summary, this work highlights the tailoring of MIC electrolytes that enable high-performance SSBs. We introduced MICs as self-supporting polymer electrolyte membranes, eliminating the need for additional organic liquid electrolytes during cell assembly. MICs are composed of a charged rigid-rod ionic polymer, poly-2,2′-disulfonyl-4,4′-benzidine terephthalamide (PBDT), along with mobile ions derived from ionic liquids, lithium salts, and functional additives. The strong associative interactions between PBDT and mobile ions create a highly adaptable system with outstanding mechanical strength, high ionic conductivity, and excellent electrochemical stability across a broad temperature range. Optimized MIC electrolytes demonstrate high ionic conductivity (3.21 mS cm^–1^ at 60 °C), a wide electrochemical stability window (5 V vs Li|Li^+^ based on LSV), and superior mechanical properties (tensile strength of 6.3 MPa, elastic modulus of 450 MPa). Additionally, MICs support stable cycling in NMC811||Li metal cells, achieving an initial specific discharge capacity of 212 mAh g^–1^ and retaining 93% of capacity after 100 cycles at 2.8–4.4 V, C/3, and 60 °C. These findings highlight MICs as a promising electrolyte platform for next-generation high-voltage solid-state lithium batteries and other advanced energy storage applications.

## Supplementary Material



## Data Availability

The data supporting the findings of this study are provided in the main article and its Supporting Information.

## Notes

Thermogravimetric analysis (TGA) of individual components and isothermal TGA of the Gen 2 polymer electrolyte; comparative table of polymer electrolytes; extended cycling performance data; additional XPS analyses; and comparison table with literature reports (PDF)

## References

[1] Mu J. (2024). Solid-state
polymer electrolytes in lithium batteries: latest progress
and perspective. Polym. Chem..

[2] Song Z. (2023). A reflection on polymer
electrolytes for solid-state lithium metal
batteries. Nat. Commun..

[3] An Y., Han X., Liu Y., Azhar A., Na J., Nanjundan A. K., Wang S., Yu J., Yamauchi Y. (2022). Progress in
Solid Polymer
Electrolytes for Lithium-Ion Batteries and Beyond. Small.

[4] Cabañero
Martínez M. A., Boaretto N., Naylor A. J., Alcaide F., Salian G. D., Palombarini F., Ayerbe E., Borras M., Casas-Cabanas M. (2022). Are Polymer-Based Electrolytes Ready for High-Voltage
Lithium Battery Applications? An Overview of Degradation Mechanisms
and Battery Performance. Adv. Energy Mater..

[5] Hatzell K. B. (2020). Challenges in Lithium Metal Anodes for Solid-State Batteries. ACS Energy Lett..

[6] Baskoro F., Wong H. Q., Yen H. J. (2019). Strategic Structural
Design of a
Gel Polymer Electrolyte toward a High Efficiency Lithium-Ion Battery. ACS Appl. Energy Mater..

[7] Wang Y., Zanelotti C. J., Wang X., Kerr R., Jin L., Kan W. H., Dingemans T. J., Forsyth M., Madsen L. A. (2021). Solid-state rigid-rod polymer composite electrolytes with nanocrystalline
lithium ion pathways. Nat. Mater..

[8] Yu Z., He Y., Wang Y., Madsen L. A., Qiao R. (2017). Molecular Structure
and Dynamics of Ionic Liquids in a Rigid-Rod Polyanion-Based Ion Gel. Langmuir.

[9] Wang Y. (2019). Double helical conformation and extreme rigidity in
a rodlike polyelectrolyte. Nat. Commun..

[10] Bostwick J. E. (2022). Ionic interactions control
the modulus and mechanical properties
of molecular ionic composite electrolytes. J.
Mater. Chem. C.

[11] Fox R. J. (2019). Nanofibrillar Ionic Polymer Composites Enable High-Modulus Ion-Conducting
Membranes. ACS Appl. Mater. Interfaces.

[12] Wang Y. (2016). Highly Conductive and
Thermally Stable Ion Gels with Tunable Anisotropy
and Modulus. Adv. Mater..

[13] Bostwick J. E. (2020). Ion Transport and Mechanical
Properties of Non-Crystallizable Molecular
Ionic Composite Electrolytes. Macromolecules.

[14] Yu D., Pan X., Bostwick J. E., Zanelotti C. J., Mu L., Colby R. H., Lin F., Madsen L. A. (2021). Room Temperature to 150 °C Lithium Metal Batteries
Enabled by a Rigid Molecular Ionic Composite Electrolyte. Adv. Energy Mater..

[15] Forsyth M., Porcarelli L., Wang X., Goujon N., Mecerreyes D. (2019). Innovative
Electrolytes Based on Ionic Liquids and Polymers for Next-Generation
Solid-State Batteries. Acc. Chem. Res..

[16] Yu D., Zanelotti C. J., Fox R. J., Dingemans T. J., Madsen L. A. (2021). Solvent-Cast Solid
Electrolyte Membranes Based on a
Charged Rigid-Rod Polymer and Ionic Liquids. ACS Appl. Energy Mater..

[17] Matsuda Y., Morita M., Yamada K., Hirai K. (1985). Characteristics of
Sulfolane-Based Electrolytes for Rechargeable Lithium Batteries. J. Electrochem. Soc..

[18] Cai H., Jing H., Zhang X., Shen M., Wang Q. (2017). Improving
High-Voltage Performance of Lithium-Ion Batteries with Sulfolane as
an Electrolyte Additive. J. Electrochem. Soc..

[19] Wang B. (2022). LiNi0.5Mn1.5O4-Hybridized
Gel Polymer Cathode and Gel Polymer Electrolyte
Containing a Sulfolane-Based Highly Concentrated Electrolyte for the
Fabrication of a 5 V Class of Flexible Lithium Batteries. ACS Omega.

[20] Wang S. (2019). High-Voltage Sulfolane Plasticized UV-Curable Gel Polymer
Electrolyte. Polymers.

[21] Evans J., Vincent C. A., Bruce P. G. (1987). Electrochemical
measurement of transference
numbers in polymer electrolytes. Polymer.

[22] Liao B., Li H., Xu M., Xing L., Liao Y., Ren X., Fan W., Yu L., Xu K., Li W. (2018). Designing Low Impedance
Interface Films Simultaneously on Anode and Cathode for High Energy
Batteries. Adv. Energy Mater..

[23] Kim S., Lee T. K., Kwak S. K., Choi N. S. (2022). Solid Electrolyte
Interphase Layers by Using Lithiophilic and Electrochemically Active
Ionic Additives for Lithium Metal Anodes. ACS
Energy Lett..

[24] Liu S. (2020). LiFSI
and LiDFBOP Dual-Salt Electrolyte Reinforces the Solid Electrolyte
Interphase on a Lithium Metal Anode. ACS Appl.
Mater. Interfaces.

[25] Yang T. (2019). Lithium bisoxalatodifluorophosphate (LiBODFP) as a multifunctional
electrolyte additive for 5 V LiNi0.5Mn1.5O4-based lithium-ion batteries
with enhanced electrochemical performance. J.
Mater. Chem. A.

[26] Deng X. (2023). Tailored interface composition
improves the integrity of electrode/electrolyte
interphases for high-voltage Ni-rich lithium metal batteries in a
sulfolane-based electrolyte. Chem. Eng. J..

[27] Lu D., Xu G., Hu Z., Cui Z., Wang X., Li J., Huang L., Du X., Wang Y., Ma J., Lu X. (2019). Deciphering
the Interface of a High-Voltage (5 V-Class)
Li-Ion Battery Containing Additive-Assisted Sulfolane-Based Electrolyte. Small Methods.

[28] Han J. G. (2017). Interfacial Architectures
Derived by Lithium Difluoro­(bisoxalato)
Phosphate for Lithium-Rich Cathodes with Superior Cycling Stability
and Rate Capability. ChemElectrochem.

[29] Donzelli M. (2022). On the Surface Modification
of LLZTO with LiF via a Gas-Phase Approach
and the Characterization of the Interfaces of LiF with LLZTO as Well
as PEO+LiTFSI. Materials.

[30] Cao W. (2025). Boosting stable lithium deposition via Li3N-Enriched inorganic SEI
induced by a polycationic polymer layer. J.
Colloid Interface Sci..

[31] Wang H., Qiu X., Wang W., Jiang L., Liu H. (2019). Iron Sulfide Nanoparticles
Embedded Into a Nitrogen and Sulfur Co-doped Carbon Sphere as a Highly
Active Oxygen Reduction Electrocatalyst. Front.
Chem..

[32] Diao Y., Xie K., Xiong S., Hong X. (2012). Insights into
Li-S Battery Cathode
Capacity Fading Mechanisms: Irreversible Oxidation of Active Mass
during Cycling. J. Electrochem. Soc..

[33] Hirata K., Morita Y., Kawase T., Sumida Y. (2018). Electrochemical performance
of an ethylene carbonate-free electrolyte based on lithium bis­(fluorosulfonyl)­imide
and sulfolane. J. Power Sources.

[34] Li S. (2012). Electrochemical performances
of two kinds of electrolytes based on
lithium bis­(oxalate)­borate and sulfolane for advanced lithium ion
batteries. J. Power Sources.

[35] Li S. (2012). Composition analysis
of the solid electrolyte interphase film on
carbon electrode of lithium-ion battery based on lithium difluoro­(oxalate)­borate
and sulfolane. J. Power Sources.

[36] Zhao Q., Zhang Y., Tang F., Zhao J., Li S. (2017). Mixed Salts
of Lithium Difluoro (Oxalate) Borate and Lithium Tetrafluorobotate
Electrolyte on Low-Temperature Performance for Lithium-Ion Batteries. J. Electrochem. Soc..

[37] Liu H. Y. (2025). Functional ternary salt construction enabling an in-situ Li3N/LiF-enriched
interface for ultra-stable all-solid-state lithium metal batteries. J. Energy Chem..

[38] Lei Y., Xu X., Yin J., Xi K., Wei L., Zheng J., Wang Y., Wu H., Jiang S., Gao Y. (2024). LiF/Li3N-Rich
Electrode–Electrolyte Interfaces Enabled by Multi-Functional
Electrolyte Additive to Achieve High-Performance Li/LiNi0.8Co0.1Mn0.1O2
Batteries. Small.

[39] Ugata Y. (2021). Understanding the Reductive Decomposition of Highly Concentrated
Li Salt/Sulfolane Electrolytes during Li Deposition and Dissolution. ACS Appl. Energy Mater..

[40] He X. (2022). Understanding the role of additive in the solvation structure and
interfacial reactions on lithium metal anode. J. Mater. Chem. A.

[41] Song G., Yi Z., Su F., Xie L., Chen C. (2021). New Insights into the
Mechanism of LiDFBOP for Improving the Low-Temperature Performance
via the Rational Design of an Interphase on a Graphite Anode. ACS Appl. Mater. Interfaces.

[42] Zhang Q. (2015). Enhanced electrochemical
performance and thermal stability of LiNi
0.5 Mn 1.5 O 4 using an electrolyte with sulfolane. Phys. Chem. Chem. Phys..

[43] Ye X. (2023). An ultra-thin polymer electrolyte for 4.5 V high voltage LiCoO2 quasi-solid-state
battery. Chem. Eng. J..

[44] Hu L. (2024). In Situ Construction of LiF-Li3N-Rich Interface Contributed
to Fast
Ion Diffusion in All-Solid-State Lithium-Sulfur Batteries. ACS Nano.

[45] Howlett P. C., Brack N., Hollenkamp A. F., Forsyth M., MacFarlane D. R. (2006). Characterization
of the Lithium Surface in N-Methyl-N-alkylpyrrolidinium Bis­(trifluoromethanesulfonyl)­amide
Room-Temperature Ionic Liquid Electrolytes. J. Electrochem. Soc..

[46] Aurbach D., Weissman I., Zaban A., Chusid O. (1994). Correlation between
surface chemistry, morphology, cycling efficiency and interfacial
properties of Li electrodes in solutions containing different Li salts. Electrochim. Acta..

[47] Zhang F. (2020). Synergistic effect of sulfolane and lithium Difluoro­(oxalate)­borate
on improvement of compatibility for LiNi0.8Co0.15Al0.05O2 electrode. Electrochim. Acta..

[48] Li S., Li J., Wang P., Ding H., Zhou J., Li C., Cui X. (2024). Interface Engineering Regulation by Improving Self-Decomposition
of Lithium Salt-Type Additive using Ultrasound. Adv. Funct. Mater..

[49] Bian X., Pang Q., Wei Y., Zhang D., Gao Y., Chen G. (2018). Dual Roles of Li3N
as an Electrode Additive for Li-Excess Layered
Cathode Materials: A Li-Ion Sacrificial Salt and Electrode-Stabilizing
Agent. Chem. – A Eur. J..

[50] Madec L., Ellis L. D. (2017). Exploring Interactions between Electrodes
in Li­[Ni
x Mn y Co 1-xy]O 2/Graphite Cells through Electrode/Electrolyte Interfaces
Analysis. J. Electrochem. Soc..

[51] Yu Y. (2021). Enhanced Cycling of
Ni-Rich Positive Electrodes by Fluorine Modification. J. Electrochem. Soc..

[52] Lu Y. C., Mansour A. N., Yabuuchi N., Shao-Horn Y. (2009). Probing the
origin of enhanced stability of AlPO4 nanoparticle coated liCoO2 during
cycling to high voltages: Combined XRD and XPS studies. Chem. Mater..

[53] Sun B. (2015). At the polymer electrolyte interfaces: the role of the polymer host
in interphase layer formation in Li-batteries. J. Mater. Chem. A.

[54] Jung R. (2018). Effect of Ambient Storage on the Degradation of Ni-Rich Positive
Electrode Materials (NMC811) for Li-Ion Batteries. J. Electrochem. Soc.

[55] Pazhaniswamy S., Joshi S. A., Hou H., Parameswaran A. K., Agarwal S. (2023). Hybrid Polymer Electrolyte Encased Cathode Particles
Interface-Based Core–Shell Structure for High-Performance Room
Temperature All-Solid-State Batteries. Adv.
Energy Mater..

[56] Pei F., Huang Y., Wu L., Zhou S., Kang Q., Lin W., Liao Y., Zhang Y., Huang K., Shen Y., Yuan L. (2024). Multisite Crosslinked Poly­(ether-urethane)-Based Polymer
Electrolytes for High-Voltage Solid-State Lithium Metal Batteries. Adv. Mater..

[57] Gao S. (2023). Fiber-reinforced
quasi-solid polymer electrolytes enabling stable
Li-metal batteries. Mater. Adv..

[58] Zhang J. (2024). An all-in-one free-standing
single-ion conducting semi-solid polymer
electrolyte for high-performance practical Li metal batteries. Energy Environ. Sci..

[59] Hou T., Qian Y., Li D., Xu B., Huang Z., Liu X., Wang H., Jiang B., Xu H., Huang Y. (2023). Electronegativity-Induced
Single-Ion Conducting Polymer Electrolyte for Solid-State Lithium
Batteries. Energy Environ. Mater..

[60] Xin C., Wen K., Guan S., Xue C., Wu X., Li L., Nan C.-W. (2022). A Cross-Linked Poly­(Ethylene Oxide)-Based Electrolyte
for All-Solid-State Lithium Metal Batteries With Long Cycling Stability. Front. Mater..

[61] Li G. (2022). Achieving a Highly Stable
Electrode/Electrolyte Interface for a Nickel-Rich
Cathode via an Additive-Containing Gel Polymer Electrolyte. ACS Appl. Mater. Interfaces.

[62] Yu J., Zhou G., Li Y., Wang Y., Chen D., Ciucci F. (2023). Improving Room-Temperature
Li-Metal Battery Performance
by In Situ Creation of Fast Li+ Transport Pathways in a Polymer-Ceramic
Electrolyte. Small.

[63] Chen Y.-H., Hsieh Y.-C., Liu K. L., Wichmann L., Thienenkamp J. H., Choudhary A., Bedrov D., Winter M., Brunklaus G. (2022). Green Polymer Electrolytes Based on Polycaprolactones for Solid-State
High-Voltage Lithium Metal Batteries. Macromol.
Rapid Commun..

[64] Li Y. (2021). A High-Voltage Hybrid
Solid Electrolyte Based on Polycaprolactone
for High-Performance all-Solid-State Flexible Lithium Batteries. ACS Appl. Energy Mater..

