# Supplementary material for: Molecular Ionic Composite Polymer Electrolytes for High-Voltage Batteries
Source: ACS Appl Mater Interfaces. 2025 Jun 15;17(25):36639–49. doi: 10.1021/acsami.5c04566 (PMC12203459; doi:10.1021/acsami.5c04566)
Supplement: Supplementary file 1 [file am5c04566_si_001.pdf]

Supporting information
Molecular ionic composite polymer electrolytes for high-voltage batteries

Jungki Min[1], Zhaohui Liang[1], Nicholas F. Pietra[1,2], Callum Connor[1], Louis A. Madsen[1,2], Feng Lin[1,2,3*]

[1] Department of Chemistry, Virginia Tech, Blacksburg, VA 24061, USA

[2] Macromolecules Innovation Institute, Virginia Tech, Blacksburg, Virginia 24061, United States

[3] Department of Materials Science and Engineering, Virginia Tech, Blacksburg, VA 24061, USA

[*]Corresponding: fenglin@vt.edu

Table of Contents

Figure S1. .... S3
Figure S2. .... S5
Figure S3. .... S6
Figure S4. .... S7
Figure S5. .... S8
Figure S6. .... S9
Figure S7. .... S10
Table S1. .... S11
References S13

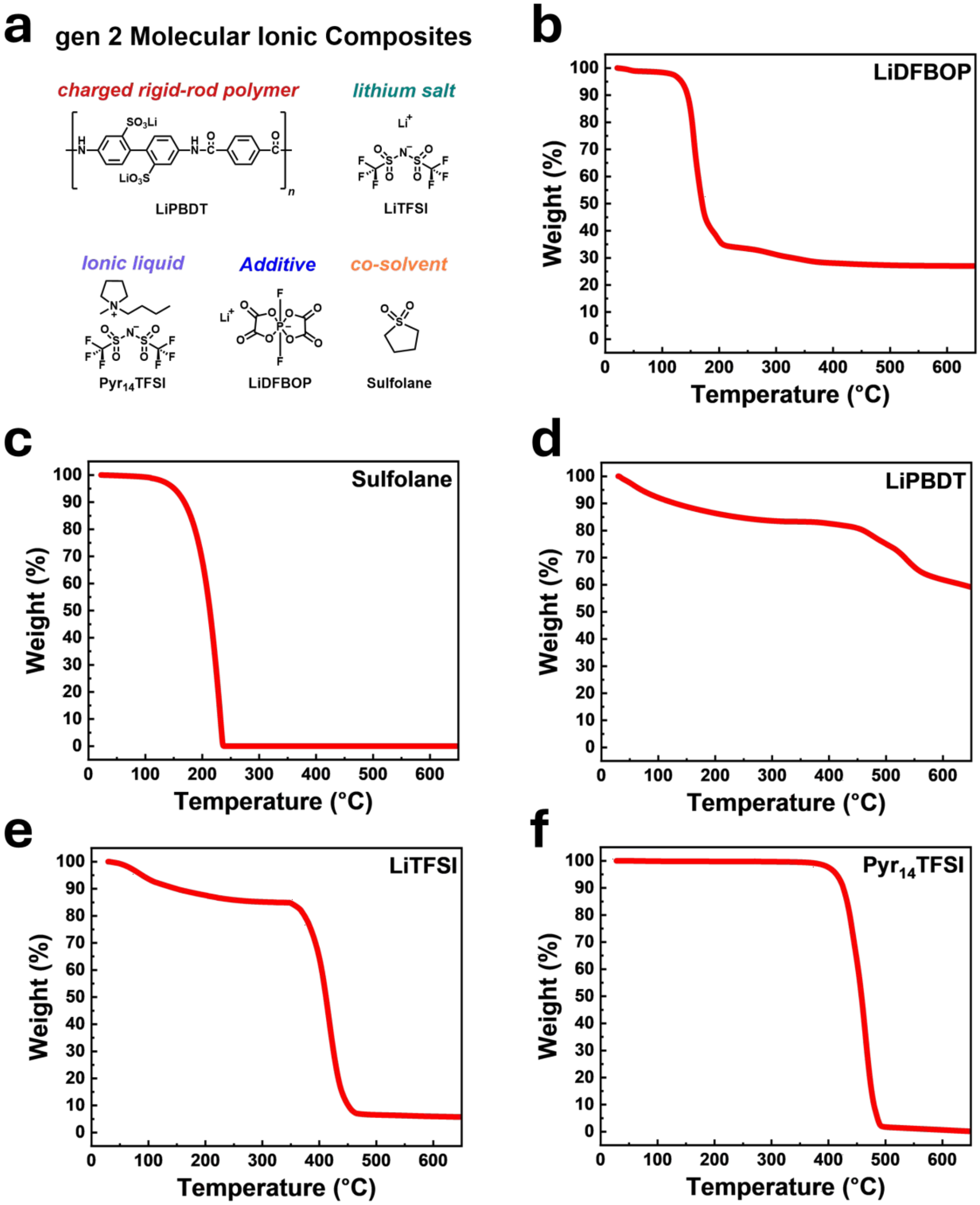

Figure S1. Thermogravimetric analysis (TGA) of individual components in gen 2 MIC electrolyte. (a) Schematic of the gen 2 MIC formulation, comprising a charged rigid-rod polymer (PBDT), lithium salt (LiTFSI), ionic liquid ($Pyr_{14}TFSI$), co-solvent (sulfolane), and additive (LiDFBOP). (b–f) TGA profile of (b) LiDFBOP, (c) sulfolane, (d) LiPBDT, (e) LiTFSI, and (f) $Pyr_{14}TFSI$. TGA was conducted under a dry N_2 atmosphere with a 10 °C min^{-1} heating rate up to 650 °C.

LiDFBOP begins to decompose near 150 °C, and sulfolane exhibits significant weight loss starting around 200 °C.

The composite gen 2 MIC membrane shows a gradual weight loss totaling ~5% below 200 °C (**Main text Figure 1e**), which can be attributed to the thermal degradation of sulfolane and LiDFBOP. In contrast, the PBDT polymer and ionic liquid display higher thermal stability. These data highlight the thermally robust nature of the MIC polymer matrix and support the observed TGA profile of the MIC membrane.

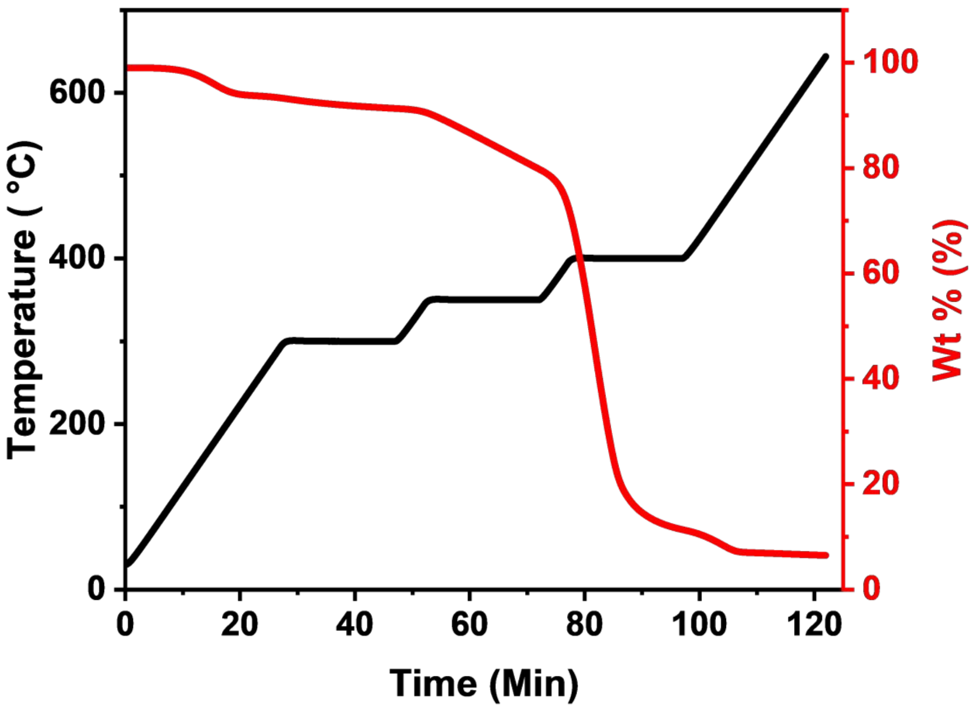

Figure S2. Thermogravimetric analysis (TGA) of gen 2 MIC electrolyte with temperature hold at 300, 250, and 400 °C. TGA was conducted under a dry N_2 atmosphere with a 10 °C min^{-1} heating rate up to 650 °C.

The isothermal steps were implemented to probe degradation behavior under extended high-temperature exposure and simulate thermal abuse conditions relevant to battery safety. The gen 2 MIC exhibited a gradual mass loss totaling ~20% below 400 °C, followed by significant decomposition above this temperature.

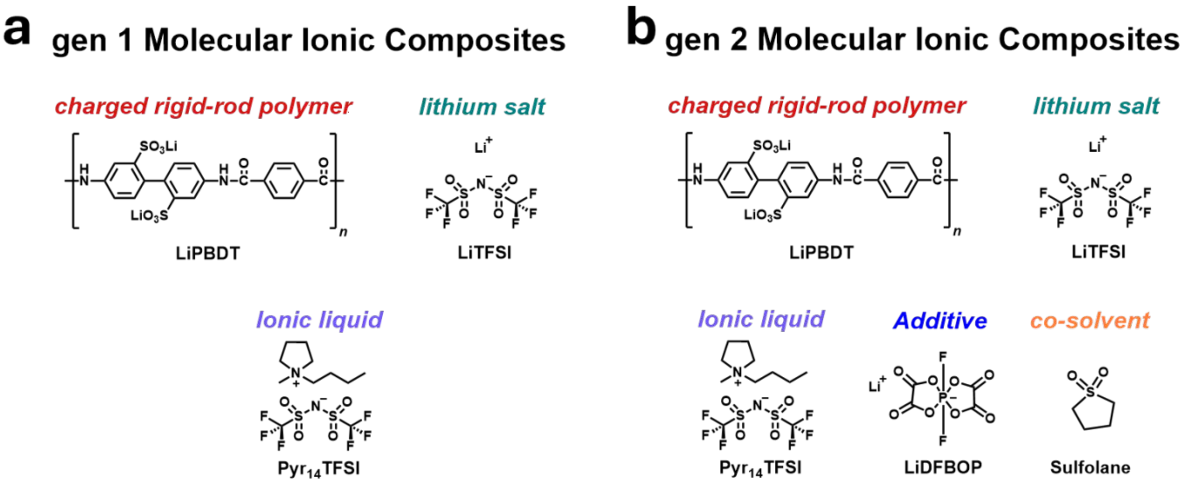

c

Property	gen 1 MIC	gen 2 MIC
Ionic Conductivity (mS cm^{-1})	2.05	3.21
Oxidation Stability (V *vs.* Li|Li^+)	3.9	5
Tensile Strength (MPa)	8	6.3
Elastic Modulus (MPa)	410	450
Diffusion Coefficient (10^{-11} m^2 s^{-1})	2.59	3.39
Capacity Retention (%)	61	93

Figure S3. Compositional and performance comparison between gen 1 and gen 2 Molecular Ionic Composite (MIC) electrolytes. (a, b) Schematic diagrams illustrating compositional differences: (a) gen 1 MIC consists of a charged rigid-rod polymer (PBDT), lithium salt (LiTFSI), and ionic liquid (Pyr_{14}TFSI); (b) gen 2 MIC adopts a multicomponent design by incorporating a co-solvent (sulfolane) and a functional additive (LiDFBOP) into the MIC matrix. (c) Summary table comparing key physical, electrochemical, and mechanical properties. Gen 2 MIC exhibits enhanced ionic conductivity and diffusion coefficient (both at 60 °C), higher oxidative stability (based on linear sweep voltammetry), comparable mechanical strength, and improved cycling performance in Li||NMC811 cells (cycled at 2.8–4.4 V, C/3, 60 °C, for 100 cycles).

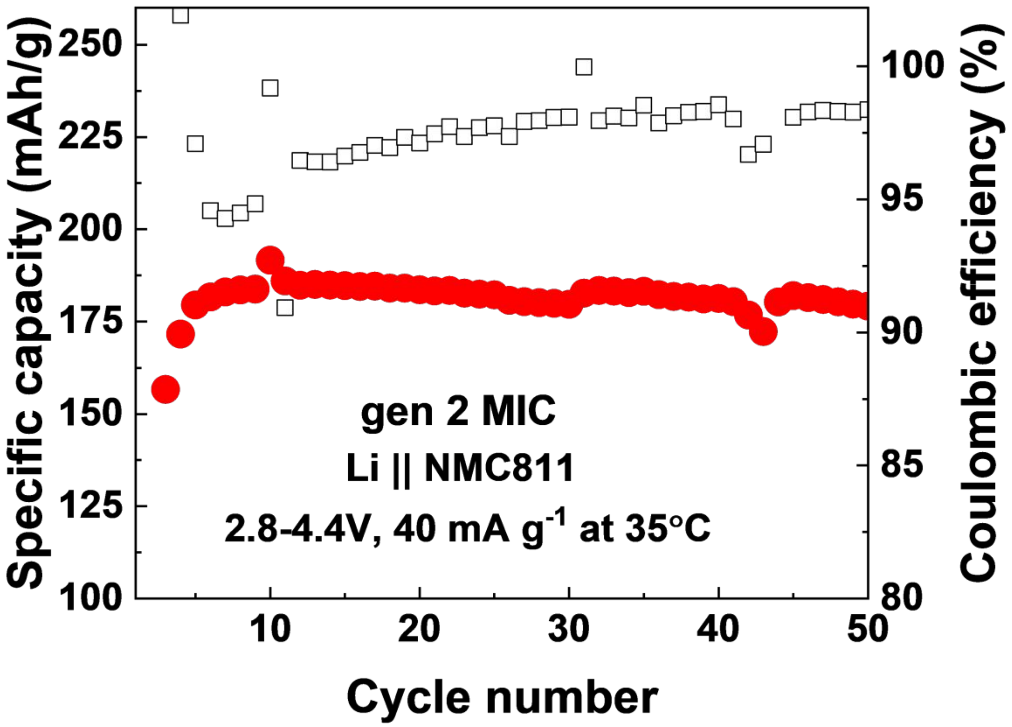

Figure S4. Cycling performance of Li|gen 2 MIC|NMC811 cell tested at 2.8–4.4 V, 40 mA g^{-1}, and 35 °C. The cell underwent two initial pre-conditioning cycles at 2.8–4.4 V, 10 mA g^{-1}, and 60 °C.

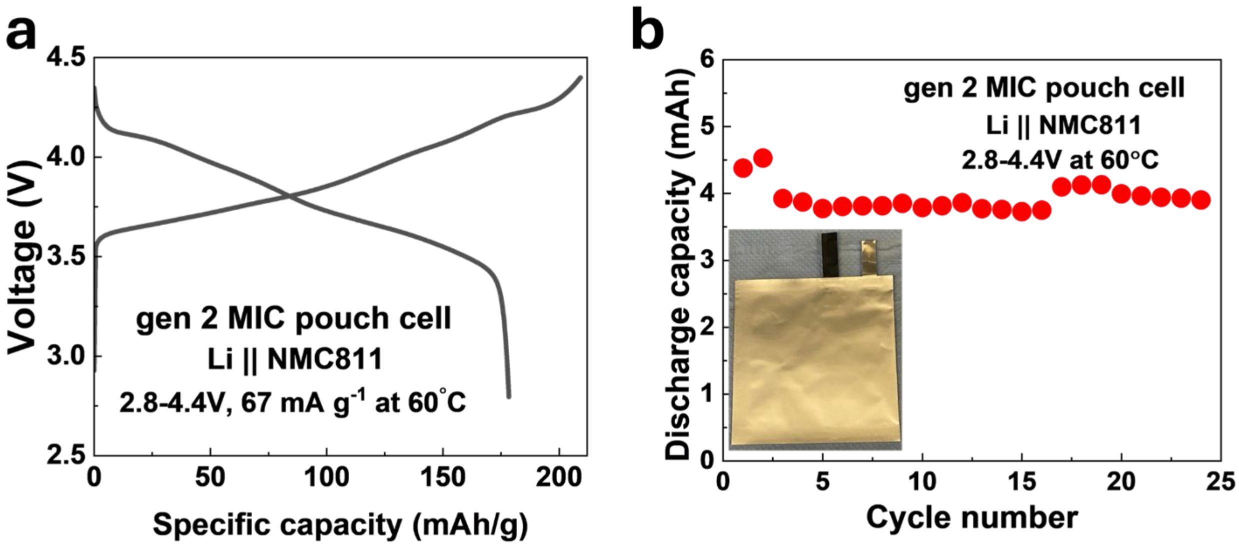

Figure S5. Electrochemical performance of a Li‖gen 2 MIC‖NMC811 single-layer pouch cell cycled between 2.8–4.4 V at 60 °C. (a) Charge–discharge voltage profile at 67 mA g^{-1} (corresponding to the third cycle in panel b). (b) Extended cycling performance. The cell underwent two initial formation cycles at 10 mA g^{-1}, followed by continuous cycling at 67 mA g^{-1}. The inset displays a photograph of the assembled pouch cell composed of a 2.8 × 2.8 cm^2 NMC811 cathode (areal capacity ≈ 0.6 mAh cm^{-2}), a 3.3 × 3.3 cm^2 Gen 2 MIC membrane (~100 µm thick), and a 50 µm-thick lithium metal anode.

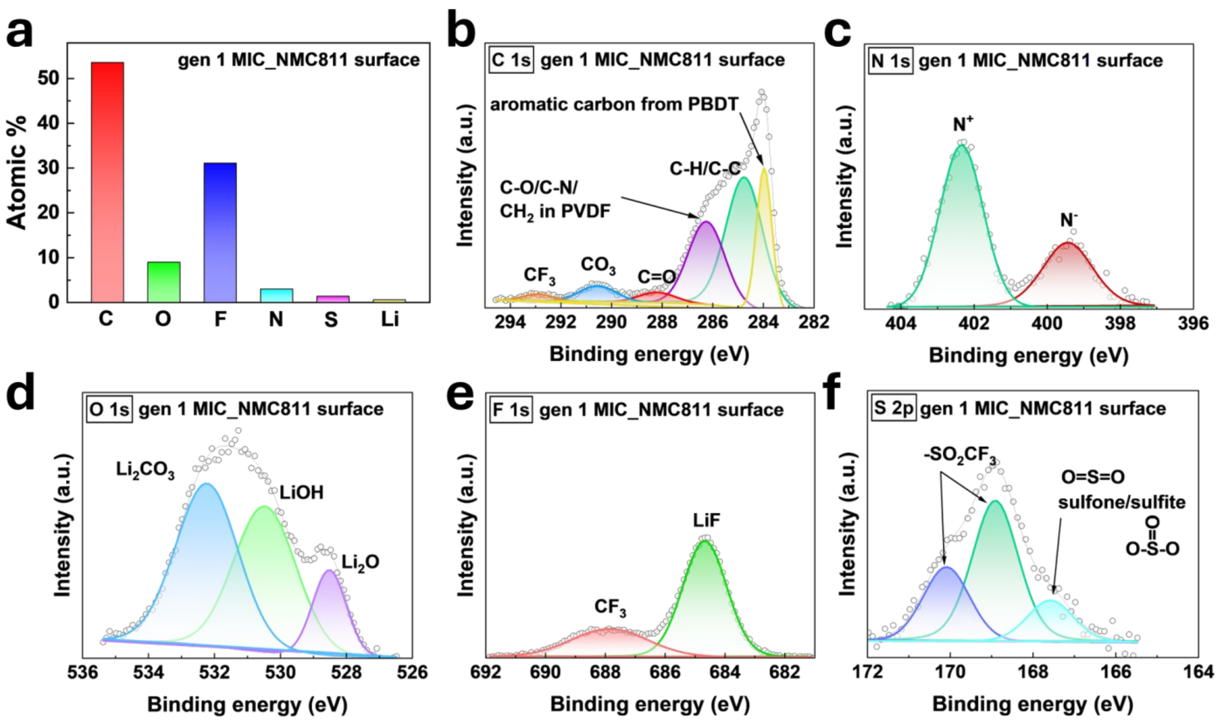

Figure S6. XPS analysis of the NMC811 surface from a Gen 1 MIC-based coin cell after electrochemical cycling (2.8–4.4 V, 67 mA g^{-1}, 200 cycles at 60 °C). (a) Atomic concentrations of detected elements on the NMC811 surface, including C (carbon), O (oxygen), F (fluorine), N (nitrogen), S (sulfur), and Li (lithium). Trace amounts of transition metals (Ni, Co, Mn) were also observed (≤1%). (b) XPS spectrum of C 1s. (c) XPS spectrum of N 1s. (d) XPS spectrum of O 1s. (e) XPS spectrum of F 1s. (f) XPS spectrum of S 2p. In the C 1s spectrum, sp^2 carbon is assigned to aromatic carbon from PBDT. In the N 1s spectrum, N^+ and N^- species are attributed to the pyrrolidinium cation (Pyr_{14}^+) and the (trifluoromethanesulfonyl)imide anion ($TFSI^-$), respectively.

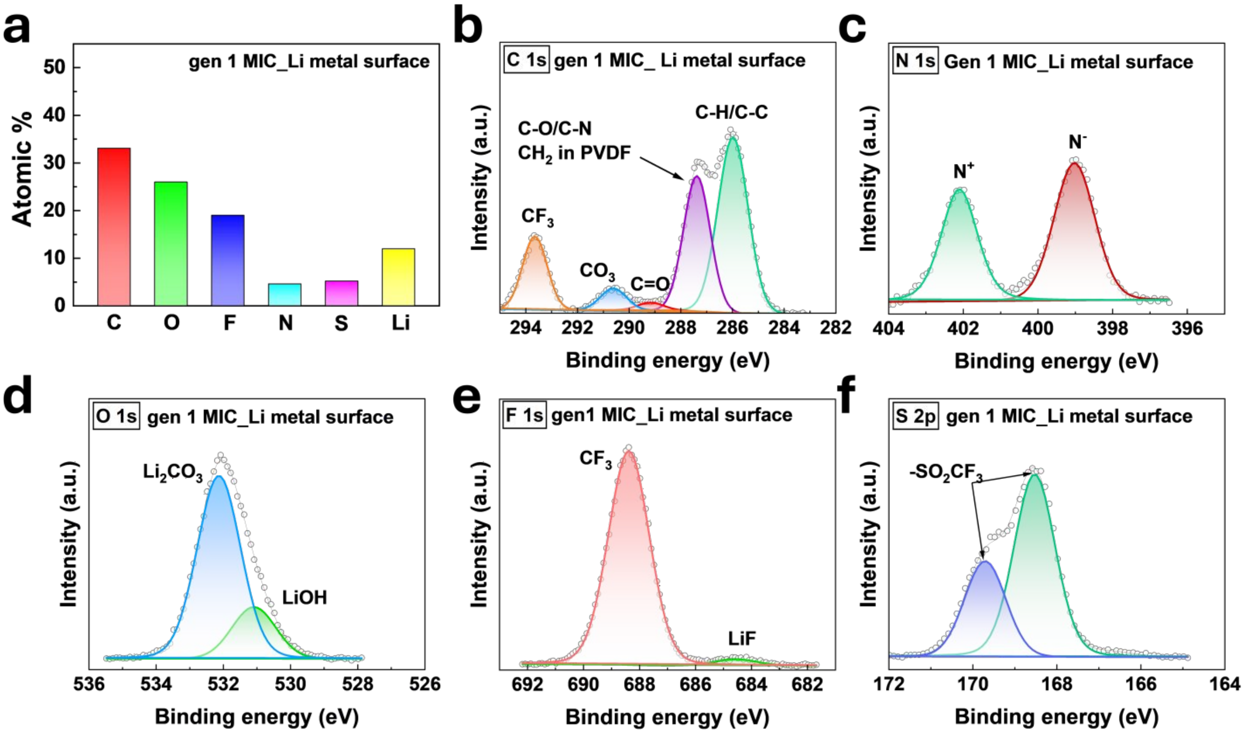

Figure S7. XPS analysis of the Li metal surface from a Gen 1 MIC-based coin cell after electrochemical cycling (2.8–4.4 V, 67 mA g^{-1}, 200 cycles at 60 °C). (a) Atomic concentrations of detected elements on the Li metal surface, including C (carbon), O (oxygen), F (fluorine), N (nitrogen), S (sulfur), and Li (lithium). (b) XPS spectrum of C 1s. (c) XPS spectrum of N 1s. (d) XPS spectrum of O 1s. (e) XPS spectrum of F 1s. (f) XPS spectrum of S 2p. In the N 1s spectrum, N^+ and N^- species are attributed to the pyrrolidinium cation (Pyr_{14}^+) and the (trifluoromethanesulfonyl)imide anion ($TFSI^-$), respectively.

Electrolytes	t_{Li+}	σ (mS cm^{-1})	Tensile strength (MPa)	Elastic modulus (MPa)	Operating voltage (V)	Specific discharge capacity (mAh g^{-1})	Operating temperature (°C)	Reference
**This work**	0.2	3.21	6.3	450	4.4	212 (at 0.33C) NMC811	60	
**Our previous work**	0.12	0.56	8	410	4.2	160 (at 1C) LFP	150	Ref[1]
**PVDF-LLZO**	0.72	0.17	15	125*	4.3	163 (at 0.3C) NMC811	25	Ref[2]
**PEU-MOF**	0.84	0.57	76.5	26*	4.3	182 (at 0.3C) NMC811	30	Ref[3]
**PPES**	0.6	1.1	14.5	133*	4.2	130 (at 0.5C) NMC811	25	Ref[4]
**PVDF-HFP-IL**	0.75	0.8	1.18	2*	4.3	209 (at 0.2C) NMC811	25	Ref[5]
**polyDOL@SEP**	0.86	0.93	50*	192*	4.3	185 (at 0.5C) NMC811	25	Ref[6]
**PEO-based**	-	0.13	10	49	4.2	120 (at 0.3C) NMC622	60	Ref[7]
**PVDF-HFP-based@PE**	0.2	6.5	126	800*	4.35	200 (at 0.5C) NMC811	25	Ref[8]
**polyDOL-PVDF-LLZTO**	0.76	1.2	-	-	4.3	140 (at 0.1C) NMC811	25	Ref[9]
**PCL-based**	0.59	0.05	-	0.1	4.3	121 (at 1C) NMC622	60	Ref[10]
**PCL-LATP**	0.58	0.036	5.37	-	4.3	120 (at 0.2C) NMC523	55	Ref[11]

Table S1. Comparison of the gen 2 MIC electrolyte with recently reported polymer electrolytes in terms of membrane physical properties, intrinsic electrochemical properties, and cell performance metrics. The selected examples involve polymer electrolytes paired with common cathode materials in lithium batteries, including $LiNi_{0.8}Mn_{0.1}Co_{0.1}O_2$ (NMC811), $LiFePO_4$ (LFP), $LiNi_{0.6}Mn_{0.2}Co_{0.2}O_2$ (NMC622), and $LiNi_{0.5}Mn_{0.2}Co_{0.3}O_2$ (NMC523). The corresponding cathode material used in each study is indicated in the table. The data are visualized in **Figure 6** of the main text. t_{Li^+} denotes the lithium-ion transference number, and σ represents ionic conductivity (values are from the original reference; "-" indicates not reported). Tensile strength and elastic modulus values are cited from references when available or estimated from reported stress–strain curves; asterisks (*) denote estimated values. Specific discharge capacities are provided at the stated C-rates, with 1C defined as 200 mA g^{-1} for NMC811, 180 mA g^{-1} for

NMC622, 160 mA g^{-1} for NMC523, and 170 mA g^{-1} for LFP. Where available, capacities were used as reported in the original references; otherwise, values were estimated based on the presentation of experimental data.

References

1. Yu, D. *et al.* Room Temperature to 150 °C Lithium Metal Batteries Enabled by a Rigid Molecular Ionic Composite Electrolyte. *Adv Energy Mater* **11**, 2003559 (2021).

2. Pazhaniswamy, S., Joshi, S. A., Hou, H., Parameswaran, A. K. & Agarwal, S. Hybrid Polymer Electrolyte Encased Cathode Particles Interface-Based Core–Shell Structure for High-Performance Room Temperature All-Solid-State Batteries. *Adv Energy Mater* **13**, 2202981 (2023).

3. Pei, F. *et al.* Multisite Crosslinked Poly(ether-urethane)-Based Polymer Electrolytes for High-Voltage Solid-State Lithium Metal Batteries. *Advanced Materials* **36**, 2409269 (2024).

4. Gao, S. *et al.* Fiber-reinforced quasi-solid polymer electrolytes enabling stable Li-metal batteries. *Mater Adv* **4**, 3452–3460 (2023).

5. Zhang, J. *et al.* An all-in-one free-standing single-ion conducting semi-solid polymer electrolyte for high-performance practical Li metal batteries. *Energy Environ Sci* **17**, 7119–7128 (2024).

6. Hou, T. *et al.* Electronegativity-Induced Single-Ion Conducting Polymer Electrolyte for Solid-State Lithium Batteries. *Energy & Environmental Materials* **6**, e12428 (2023).

7. Xin, C. *et al.* A Cross-Linked Poly(Ethylene Oxide)-Based Electrolyte for All-Solid-State Lithium Metal Batteries With Long Cycling Stability. *Front Mater* **9**, (2022).

8. Li, G. *et al.* Achieving a Highly Stable Electrode/Electrolyte Interface for a Nickel-Rich Cathode via an Additive-Containing Gel Polymer Electrolyte. *ACS Appl Mater Interfaces* **14**, 36656–36667 (2022).

9. Yu, J. *et al.* Improving Room-Temperature Li-Metal Battery Performance by In Situ Creation of Fast Li+ Transport Pathways in a Polymer-Ceramic Electrolyte. *Small* **19**, 2302691 (2023).

10. Chen, Y. H. *et al.* Green Polymer Electrolytes Based on Polycaprolactones for Solid-State High-Voltage Lithium Metal Batteries. *Macromol Rapid Commun* **43**, 2200335 (2022).

11. Li, Y. *et al.* A High-Voltage Hybrid Solid Electrolyte Based on Polycaprolactone for High-Performance all-Solid-State Flexible Lithium Batteries. *ACS Appl Energy Mater* **4**, 2318–2326 (2021).
